# Taxonomic and Functional Assessment of Microbial Communities in Urban Runoff-Contaminated Mangrove Sediments

**DOI:** 10.1007/s00284-026-04910-5

**Published:** 2026-04-16

**Authors:** João Vitor Wagner Ordine, Franciene Rabiço Oliveira, Jonatã Bortolucci, Lívia Soares Zaramela, María-Eugenia Guazzaroni

**Affiliations:** 1 Department of Biochemistry, Ribeirão Preto Medical School, University of São Paulo, Ribeirão Preto, SP Brazil; 2 Department of Biology, Faculty of Philosophy, Sciences and Letters of Ribeirão Preto, University of São Paulo, Av. Bandeirantes, 3.900, Ribeirão Preto, SP 14049-901 Brazil; 3 Department of Chemistry, Faculty of Philosophy, Sciences and Letters of Ribeirão Preto, University of São Paulo, Ribeirão Preto, 14049-900 Brazil

## Abstract

Mangroves are vital coastal ecosystems that host diverse microbial communities essential to ecological function and nutrient cycling. Yet, urbanization and pollution are accelerating their degradation. This study explored the sediment microbiota from two impacted mangroves in São Sebastião, the southeastern Brazilian coast, Araçá Bay, and Colhereiro, through microbiota profiling, sediment chemical analysis, and the isolation of native bacteria capable of growing under the exposure of urban landfill leachate, a major pollutant affecting these fragmented environments. Among the two mangroves, Colhereiro exhibited pronounced signatures of chronic urban pollution, including elevated macronutrients and enrichment of sulfur-metabolizing bacteria and *Arcobacter*, a genus associated with fecal contamination and environmental pollution. Genomic DNA from bacterial isolates showing robust growth on leachate residue was sequenced, and genome mining was conducted to evaluate their functional capabilities. The results offer novel insights into the taxonomic and functional diversity of mangrove microbiota in São Sebastião and underscore their potential for use in bioremediation and environmental restoration of polluted coastal ecosystems.

**Supplementary Information:**

The online version contains supplementary material available at 10.1007/s00284-026-04910-5.

## Introduction

Mangrove forests are highly productive coastal wetlands that provide a wide range of essential ecosystem services, including carbon sequestration, shoreline stabilization, biodiversity conservation, and the filtration of anthropogenic pollutants [1–3]. Despite their ecological and socio-economic importance, these ecosystems have experienced extensive degradation due to deforestation, pollution, and urban expansion [4]. Brazil hosts approximately 1.4 million hectares of mangroves, accounting for 9% of the global mangrove coverage [5]. However, on the northern coast of the state of São Paulo, mangrove habitats are naturally restricted due to the region’s steep topography and limited coastal plains. These fragmented ecosystems, primarily located within urbanized areas, are further threatened by intensive human activities such as oil exploration, harbor operations, and tourism, which exert significant environmental pressure [6]. Consequently, the mangroves of São Sebastião, a coastal municipality in São Paulo State, are highly vulnerable to habitat loss and contamination, particularly from urban-derived pollutants such as landfill leachate, which can profoundly impact their ecological integrity and long-term resilience [7, 8]. This region is especially concerning, as its remaining mangrove fragments are heavily polluted with urban contaminants, fragmented, or affected by harbor activities, with no pristine areas left that allow reliable ecological comparisons [8].

Mangrove microbial communities play a fundamental role in maintaining ecosystem resilience and productivity by driving essential biogeochemical processes, including carbon sequestration, organic matter degradation, and nutrient cycling [9]. These microorganisms are well adapted to the fluctuating environmental conditions of mangrove ecosystems, where variations in salinity, water levels, ultraviolet radiation, and sediment anoxia shape their functional diversity [10, 11]. Notably, sulfate-reducing, nitrogen-fixing, and phosphate-solubilizing bacteria are abundant in mangrove sediments, facilitating the transformation and availability of key nutrients such as sulfur, nitrogen, and phosphorus [12]. Due to these metabolic capabilities, mangrove-associated microbes have been widely recognized for their potential in biotechnology, particularly in bioremediation [13–16]. Previous studies have demonstrated the capacity of mangrove bacteria to degrade hydrocarbons, remediate heavy metal contamination, and support plant growth through nutrient mobilization [13, 14]. Notably, the use of indigenous microbial consortia has shown promising results in in situ mangrove decontamination, often outperforming the introduction of exogenous bacteria. Indigenous microbes are already adapted to the unique challenges of mangrove bioremediation, including the need for optimal environmental conditions and competition with native degradative microbiota [3, 4, 13]. Therefore, a deeper understanding of native microbial communities is essential for the development of site-specific bioremediation strategies, which are critical for the long-term conservation and restoration of these vulnerable ecosystems.

Despite their ecological and biotechnological significance, studies characterizing the microbial communities of highly impacted Brazilian mangroves remain scarce. In particular, few studies have performed a taxonomic and functional analysis on the bacterial communities of São Paulo’s mangroves, and no similar studies were performed in those environments in the São Sebastião municipality. Thus, we characterized the sediment-associated microbial communities of two polluted and fragmented mangrove areas in São Sebastião. We employed microbiota profiling and isolated native bacteria to assess their taxonomic and functional diversity. Additionally, we investigated the ability of bacterial isolates to grow under exposure of urban landfill leachate, a major pollutant affecting these fragmented environments. Isolates with the most expressive growth on leachate residue were sequenced, and genome mining was performed to further explore their functional potential. Our findings provide novel insights into the composition and adaptive strategies of mangrove-associated microbiota in São Sebastião and highlight their prospective relevance for future bioremediation studies and environmental restoration efforts, particularly in urbanized and contaminated coastal ecosystems.

## Materials and Methods

### Studied Area and Sample Collection

The samples in this study were collected from two environmentally impacted mangrove fragments located in the municipality of São Sebastião, São Paulo state, on the southeastern coast of Brazil. These fragments, namely Araçá Bay and Colhereiro, are situated along a stretch of coastline that has undergone significant anthropogenic transformation due to urban expansion, industrial activity, and coastal infrastructure development [6–8]. Araçá Bay (approximate area: 1.99 ha; coordinates: 23°48′30″S, 45°23′51″W) is part of a 50-hectare embayment characterized by a complex mosaic of interacting ecosystems, including sandy beaches, rocky shores, tidal mudflats, and patches of mangrove vegetation. Although once a more extensive mangrove-dominated open roadstead, Araçá has been highly fragmented due to ongoing land-use pressures [6–8]. Notably, the installation and expansion of the São Sebastião Port, and persistent dredging have physically transformed the bay and reduced the mangrove cover [6–8]. Despite this, Araçá Bay remains ecologically significant, serving as an important breeding and feeding ground for aquatic organisms [1]. The area is also vital for local artisanal fisheries and has been highlighted as a conservation priority due to its high biodiversity and social importance [6–8]. The bay experiences semi-diurnal microtidal regimes (ranging from -0.04 m to 2.06 m), a mean annual rainfall of 2,600 mm, and average temperatures around 20 °C [6–8].

Colhereiro, by contrast, is a much smaller mangrove fragment (approximate area: 0.45 ha; coordinates: 23°48′46″S, 45°24′29″W) that developed secondarily following anthropogenic modifications to the landscape. Historical aerial imagery indicates that this site was previously a sandy coastal area, with mangrove colonization occurring post-disturbance due to ecological succession [1, 8]. Located near the São Sebastião ferry terminal, the Colhereiro mangrove is heavily exposed to urban pressures, including illegal waste disposal, intense pedestrian traffic, the proliferation of invasive plant species, and a general lack of protective infrastructure [1, 8]. According to the São Paulo State Forest Foundation (2014), Colhereiro is among the priority mangrove sites for conservation in São Sebastião, alongside Araçá and other mangroves in the region, due to its ecological roles and degraded condition [17].

**Fig. 1:**
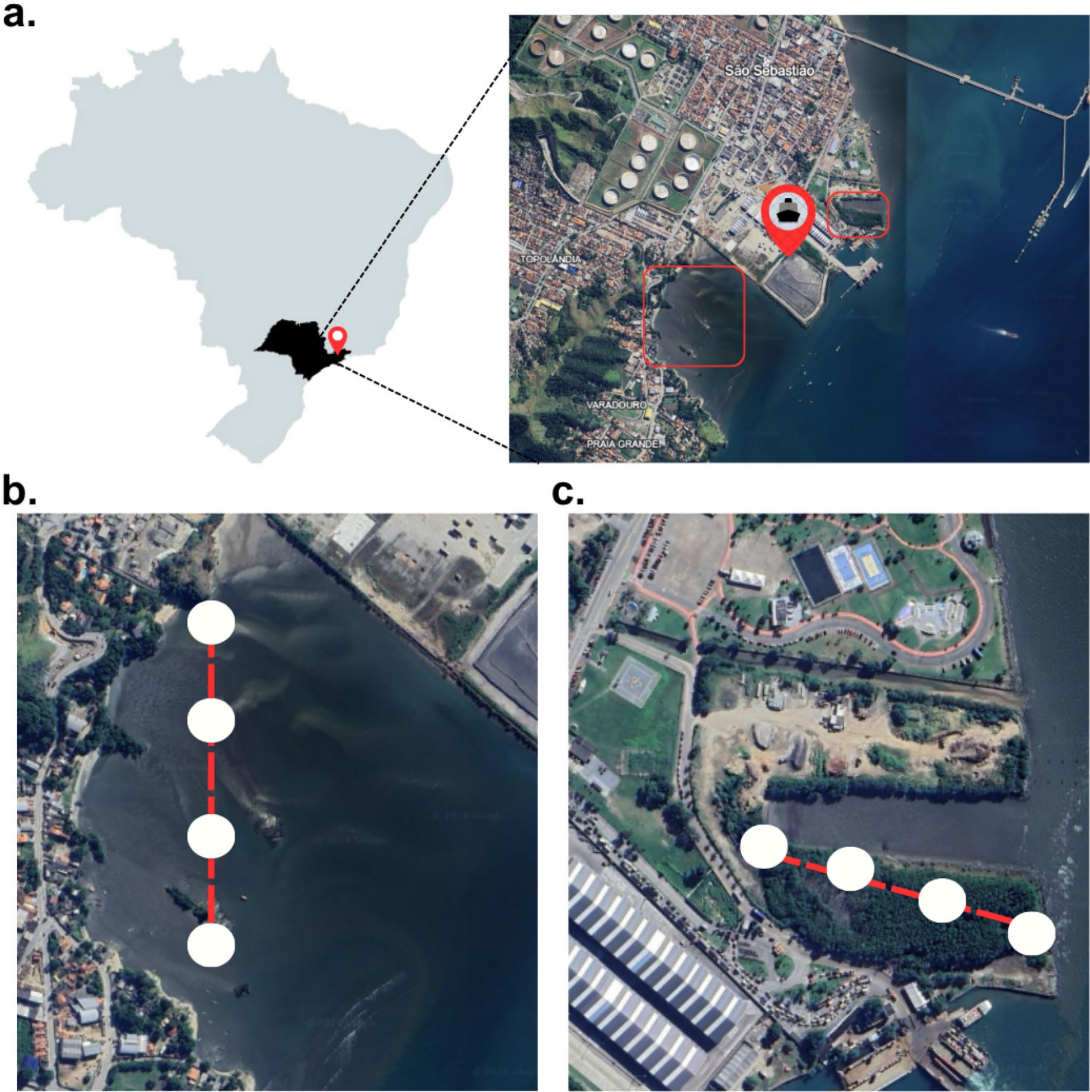
Map of studied mangrove fragments in São Sebastião, São Paulo, Brazil. **A.** Schematic representation of the Brazil map (in gray), with São Paulo state highlighted in black. A red pin in the map indicates the approximate location of the São Sebastião municipality. The scheme was created in MapChart (https://www.mapchart.net/index.html). Part of the São Sebastião municipality was zoomed in. The map was taken using Google Maps and Google Earth Pro (https://www.google.cl/maps; accessed on 9 November 2023). Red squares denote the mangrove fragments where soil samples were collected: the larger square on the left represents the Araçá Bay mangrove fragment, and the smaller square on the right represents the Colhereiro mangrove fragment. The red pin between both fragments indicates the location of São Sebastião’s port. Figures **B.** (Araçá Bay) and **C.** (Colhereiro) represent the transect (dashed red line) performed for sample collection. In each site, four independent samples were collected, represented by white dots (Color figure online)

During low tide, four sediment samples (approximately 40 g each) were collected from each mangrove fragment along a 60-m transect, with sampling points spaced every 20 m (Fig. 1). Sediments were extracted using a sterile steel corer to a depth of 10 cm, transferred to sterile polypropylene tubes, and immediately placed in a portable cooler at 4 °C. Samples were transported to the laboratory within two hours for immediate processing or stored at − 80 °C for subsequent environmental DNA (eDNA) extraction, chemical analyses, and bacterial isolation.

### Chemical Analysis of Sediments

The soil sample submitted for chemical analysis at Ribersolo (Ribeirão Preto, Brazil) was prepared by pooling approximately 25 g of sediment from each of the four collection points within each mangrove fragment, resulting in a composite sample of about 100 g per site. The chemical analyses were performed on dried soil samples at 40 °C, according to the Agronomic Institute’s and Brazilian Agricultural Research Corporation’s [18, 19] methodologies. The following parameters were quantified: phosphate (PO4^3^**⁻**), potassium (K⁺), calcium (Ca^2^⁺), magnesium (Mg^2^⁺) and aluminum (Al^3^⁺) concentrations; base saturation (V%); pH (measured in CaCl₂); total organic matter (TOM); potential acidity (H⁺ + Al^3^⁺); cation exchange capacity (CEC); sum of exchangeable bases (BS); and aluminum saturation (m%).

### Environmental DNA Extraction and Amplicon-Based Sequencing

eDNA was extracted from 1 g of sediment collected from four independent points at each mangrove site. The extraction was performed using the DNeasy PowerSoil Kit (QIAGEN, Hilden, Germany), following the manufacturer’s protocol. The quality of the extracted eDNA was assessed using a Nanodrop One spectrophotometer (Thermo Fisher Scientific, Waltham, USA) and 0.8% agarose gel electrophoresis.

For whole-community 16S rRNA gene amplification, we used the 16S Barcoding Kit 1–24 (SQK-16S024), following the manufacturer’s instructions. Library preparation was carried out using LongAmp® Hot Start Taq 2X Master Mix (New England Biolabs), with eDNA at a final concentration of 10 ng/µL. Each sample was combined with 10 µL of its corresponding 16S barcode. The PCR amplification conditions were as follows: initial denaturation at 95 °C for 1 min, followed by 25 cycles of denaturation at 95 °C for 30 s, annealing at 55 °C for 30 s, and extension at 65 °C for 2 min, with a final extension at 65 °C for 5 min. Barcoded amplicons were purified using LoBind Eppendorf tubes and AMPure XP beads, following the recommendations in the 16S Barcoding Kit. The purified libraries were quantified with a Nanodrop One and normalized to the same concentration. All barcoded libraries were then pooled to a final concentration of 100 ng in 10 µL and submitted to ByMyCell (Ribeirão Preto, Brazil) for amplicon-based sequencing on a MinION Mk1B platform (Oxford Nanopore Technologies, Oxford, UK).

### Bacteria Isolation and Landfill Leachate Growth Screening

The urban landfill leachate used in this study was furnished by an urban waste management service located in São Paulo (São Paulo, Brazil). Prior to its application for bacterial isolation, the leachate was manually sterilized by syringe-driven filtration through a 0.22 µm membrane filter. Filtration was performed slowly and under gentle pressure to prevent membrane rupture due to the high particulate load and viscosity of the leachate. To obtain bacterial cultures for subsequent evaluation of their capacity to utilize leachate residue as a sole nutrient source, we targeted fast-growing aerobic bacteria typically associated with bioremediation in shallow sediment environments.

Approximately 1 g of sediment from each mangrove site was homogenized in 50 mL of sterile urban landfill leachate, which served as the sole nutrient source. The homogenate underwent ten-fold serial dilutions and was incubated at 30 °C with agitation at 200 rpm for 16 h. Following incubation, aliquots from various dilutions were plated on Luria–Bertani (LB) agar (Kasvi, Pinhais, Brazil) supplemented with 4 mg/mL amphotericin B (Sigma-Aldrich, St. Louis, USA) to inhibit fungal contamination. Plates were incubated overnight at 30 °C. Bacterial colonies were subsequently purified through successive streaking and selected based on distinct colony morphologies and microscopic characteristics. Pure bacterial isolates were subjected to preliminary growth curve analyses to assess their metabolic potential. Cultures were grown in M9 minimal medium supplemented with 1% glycerol as the sole carbon source and incubated at 30 °C with shaking at 200 rpm for 16 h. Cells from each culture were harvested by centrifugation (3,000 rpm, 2 min) and washed twice by resuspension in 0.9% saline solution. The washed cells were then standardized to an optical density at 600 nm (OD_600nm_) of 0.1 and inoculated into 12-well plates containing leachate residue diluted in deionized water at ratios of 1:10, 1:20, and 1:40. OD_600nm_ measurements were taken immediately after inoculation and again after 24 h of incubation. For each dilution, medium-only controls (leachate diluted in deionized water without bacterial inoculation) were included to confirm leachate sterility and used as blanks to correct OD_600nm_ readings for background absorbance. The bacterial cultures demonstrating the highest growth performance were selected for detailed growth curve analyses. These assays were conducted under the same cultivation conditions as described above, with OD_600nm_ measurements taken every two hours during the first 10 h, as well as at 24 and 30 h. Growth monitoring was performed using a VICTOR® Nivo™ Multimode microplate reader (Perkin-Elmer, Waltham, USA). All growth curve experiments were conducted with three biological replicates for each bacterial culture. Specific growth rates (μ) were calculated for each culture under all tested conditions using the linear method [20]. *Pseudomonas putida* KT2440 was employed as a negative control in these experiments, as it is a well-characterized reference bacterium originally isolated from soil and widely used in biodegradation studies due to its versatile metabolic capabilities.

### Genomic DNA Sequencing and Bacteria Identification

The genomic DNA (gDNA) from bacterial cultures with considerable growth in leachate residue was extracted using the Wizard Genomic DNA Purification Kit (Promega, Madison, USA), following the manufacturer’s recommendations. The quality assessment was performed as previously described. Following the manufacturer's recommendations, a near-full-length PCR amplification of the 16S rDNA gene was performed for each bacterial culture using GoTaq Polymerase (Promega) and the universal primer pair 27F/1492R [21]. The resulting amplicons were purified by the Wizard SV Gel and PCR Clean-Up System (Promega) and passed a quality assessment as previously described. The bacterial cultures were identified by sequencing the amplified 16S rDNA fragments using the ABI 3500xL Genetic Analyzer (Applied Biosystems, Waltham, USA). The resulting reads were trimmed based on their electropherograms, and a BLAST search was performed to determine their taxonomic identity [21]. Whole-genome sequencing was performed on the seven bacterial cultures that exhibited the highest growth capacity when incubated with leachate as the sole nutrient source. Sequencing was conducted using the Illumina NovaSeq 6000 platform at Novogene (Sacramento, USA).

### Data Processing and Bioinformatic Analyses

Processing and analysis of the whole community 16S rDNA reads were partially adapted from Ordine and collaborators (2023) [22]. Briefly, raw nanopore sequencing data underwent base-calling using Guppy Software v6.1.3 in high-accuracy mode, leveraging a graphics processing unit (GPU) for real-time base-calling [23]. Demultiplexing was performed with Porechop v2.4, using barcodes from SQK-16S024 [24]. The quality of the reads was assessed using NanoQC v0.10.0, NanoComp v1.24.2, and NanoPlot v1.44.1 from the NanoPack toolset [25]. Low-quality bases and adapter sequences were trimmed and filtered using chopper 0.9.2 based on read length and quality scores [25, 26]. Demultiplexed reads were aligned to SILVA non-redundant 16S rDNA database v138.2, using k-mer alignment with KMA v1.4.14 [27–30]. To ensure comparability between samples and avoid sequencing depth bias in downstream analyses, we performed rarefaction of all samples above 1,000 reads prior to calculating diversity metrics. Microbial community analysis was conducted in R, incorporating alpha and beta diversity metrics (phyloseq, vegan), taxonomic assignments and visualizations (phyloseq, tidyverse), and correlation analyses (corrplot, ecodist, rstatix). To ease the visualization of the most representative members of the microbial communities present in the studied mangroves, all detected genera with relative abundance above 1.5% were plotted in stacked bar charts, in which each bar represents the summed relative abundances of most representative taxa identified in that replica. All genera whose relative abundance was equal or inferior to 1.5% were combined in the “Others” category. Comparisons between the relative abundances of main taxa were performed using the Wilcoxon rank-sum test, and the resulting *p*-values were corrected for multiple comparisons using the Bonferroni method to control for type I error, with a significance level fixed at α = 5%. Alpha diversity differences were similarly tested with the Wilcoxon signed-rank test and Bonferroni correction (α = 5%). To assess beta-diversity, we first calculated Bray–Curtis dissimilarity between each pair of microbial members in a specific mangrove and then performed a principal coordinate analysis (PCoA) in R; the resulting ordination axes were annotated with the proportion of variance explained by each principal coordinate. Microbe-microbe correlations were calculated with Spearman's correlation test, and significant correlations (*p*-value < 0.05) were represented in a heatmap.

The whole genome of the selected bacterial cultures was processed with an adapted pipeline from Borelli et al., 2021 [31]. In summary, reads had their quality assessed with FastQC v0.12.1, and their quality reports were created with MultiQC v1.17. Reads with possible human contamination were removed by aligning them against the human reference genome (GRCh38) using Bowtie2 v2.4.1 (parameters: -D 20 -R 3 -N 1 -L 20 –very-sensitive-local) [32]. Overlapped paired-end reads were merged with Flash v1.2.11 to generate longer reads from fragment libraries before genome assembly [33], and then, reads were assembled de novo with Spades v3.15.5 (parameters: –merge -s), using both merged and unmerged reads [34]. Contigs with sequences shorter than 500 bp were removed using the Biostrings package in R. The overall DNA sequencing quality was assessed with QUAST v5.2.0 [35]. Genome completeness and contamination were subsequently estimated using CheckM2 v1.1.0 [36]. An additional screening for the removal of remaining adaptors was performed with Foreign Contamination Screening (FCS)-adaptor executable, that removes terminal and internal matches to an NCBI-non-redundant database of adaptors [37]. Genome annotation was performed with Prokka v1.14.6, using default parameters [38]. The ABRICATE pipeline was used to identify antibiotic resistance genes (ARGs), virulence genes (VGs), and plasmids in the selected bacterial cultures (databases: –ncbi, –card, –vfdb, and –plasmidfinder) [39–41]. The genome sequence data were uploaded to the Type Strain Genome Server (TYGS; https://tygs.dsmz.de) for taxonomic analyses on 2024–03-07 [42–48]. TYGS was used to perform both whole-genome-based phylogenomic analyses (based on Genome BLAST Distance Phylogeny, GBDP) and 16S rRNA gene-based phylogenetic inference. The trees were rooted at the midpoint and visualized with PhyD3 [49]. The type-based species clustering was performed with a 70% dDDH threshold, and a species was identified when the *d4* formula and branch support values exceeded 70%. The complete methodology for the taxonomic analysis in TYGS can be assessed in the supplementary materials. Taxonomic names were verified using the List of Prokaryotic names with Standing in Nomenclature (LPSN; accessed on 2024–03-07). An additional genome annotation using the RAST web server was performed to assess the genomic capabilities of the bacterial cultures, mining sequences putatively involved in the metabolism of aromatic compounds, nitrogen, sulfate, phosphorus, and other features that could indicate potential bioremediation applications for the isolated bacteria [50–52]. To assess how unique the features identified through genome mining were, the obtained results were compared with the reference genome of the strain *Priestia megaterium* ATCC 14581.

## Results

### Chemical Characteristics of Studied Mangroves

Notable differences were identified when analyzing the chemical parameters of the studied mangroves (Table S1). The Araçá Bay mangrove sediments presented a slightly lower pH (7.3) when compared to Colhereiro sediments (7.8). No differences were observed between sites regarding potential acidity (H⁺ + Al^3^⁺ = 9) or aluminum concentration and saturation (m%). In contrast, Colhereiro sediments exhibited substantially higher concentrations of macronutrients (PO4^3^-, Ca^2^⁺, Mg^2^⁺, and K⁺), as well as higher values for the sum of exchangeable bases (SB) and cation exchange capacity (CEC), which were approximately two-fold greater than those measured in Araçá Bay. The most pronounced difference between sites was observed in total organic matter (TOM), which was 4.7-fold higher in Colhereiro sediments compared to Araçá Bay.

### Mangrove Bacterial Communities

After demultiplexing the four independent mangrove samples for each studied site, an average number of 2,651 ± 1,264 and 2,686 ± 680 full-length raw reads were obtained for Araçá (A1-A4) and Colhereiro replicas (B1-B4), respectively. Lower-quality extremities were trimmed, and low-quality reads were removed, obtaining at the end of the quality control steps an average number of 1,738 ± 913 high-quality reads for each Araçá replica and 1,746 ± 471 for Colhereiro. Detailed sequencing metrics, including SRA accession numbers and read counts before and after quality filtering, are provided in Table S2 (see also Figures S1 and S2).

**Fig. 2:**
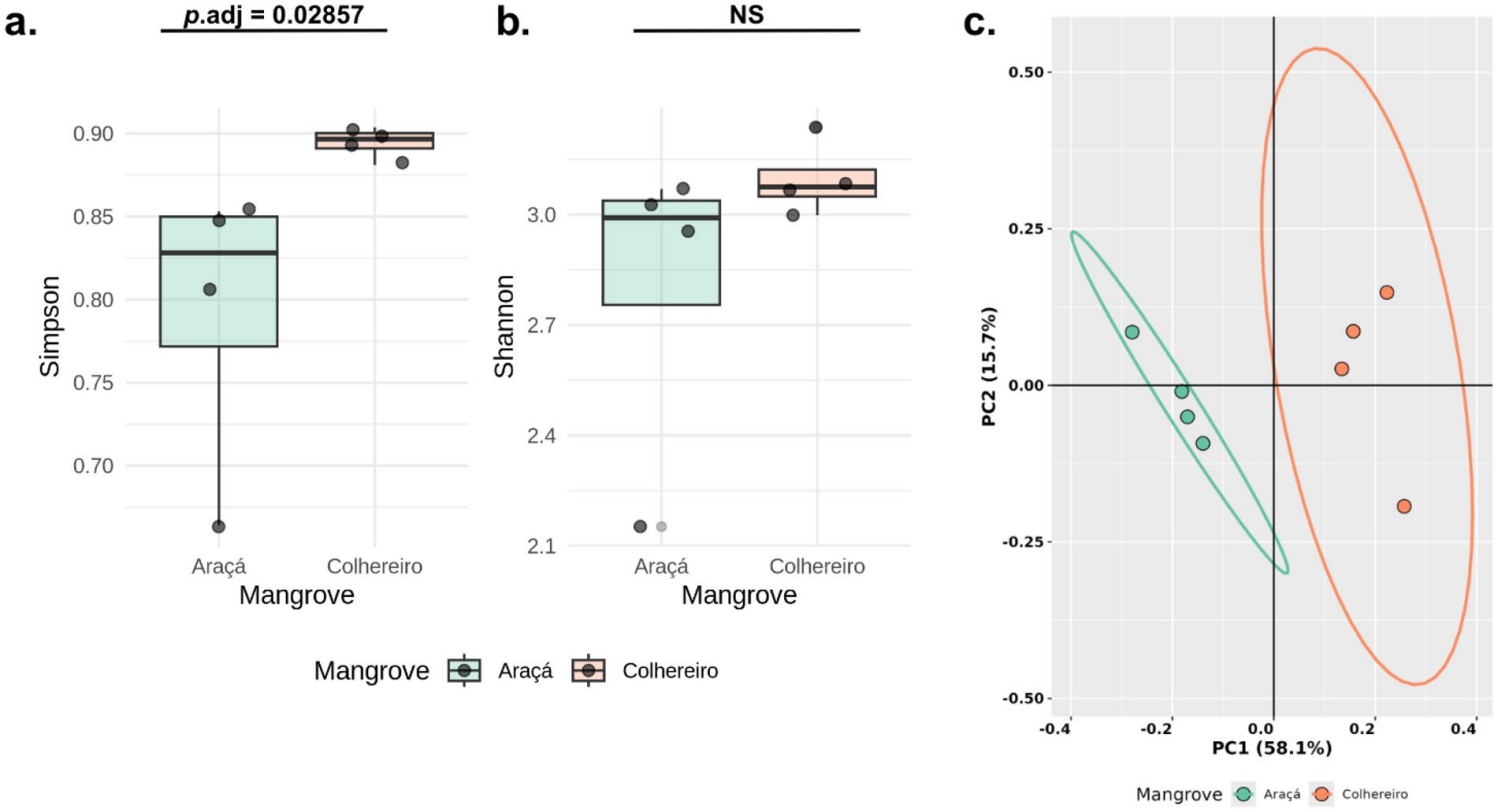
Diversity analysis of mangrove microbial communities. **(A, B).** Alpha diversity metrics represented as boxplots (**A.** Simpson and **B.** Shannon). **C.** Beta diversity visualized by Principal Coordinates Analysis (PCoA) based on Bray–Curtis dissimilarity. Each point represents a biological replicate: pink dots correspond to Araçá mangrove samples (**A1–A4**), and blue dots correspond to Colhereiro mangrove samples (**B1–B4**). Ellipses denote 95% confidence intervals for each group

The taxonomic assignment of reads against the SILVA 16S database resulted in the annotation of 89.6 ± 10.8% of Araçá reads and 92.4 ± 9.38% of Colhereiro reads. To begin the diversity analysis of the sequenced samples, high-quality reads were normalized by rarefying those above 1,000 reads and collector curves were plotted to depict the number of genera detected based on sample size, which indicates that the diversity of samples at the genus level can be represented accurately in the sequenced depth performed (Figure S3). After sample rarefaction, alpha diversity indexes were calculated for each replicate individually, which were then combined according to their corresponding mangrove groups to compare the differences in the local richness of each studied site (Figure S4 and Table S3). The Simpson index revealed a statistically significant difference in alpha diversity between the two mangroves (*p* = 0.028), with higher values observed in Colhereiro samples (Fig. 2A), indicating a more even community structure with less dominance by a few highly abundant taxa. In contrast, the Shannon index showed no significant difference between sites (Fig. 2B), reflecting comparable overall richness and accounting for the presence of both common and rare taxa. These findings suggest that although microbial communities in both mangroves are similarly rich in taxa, their internal abundance distributions differ. To assess beta diversity, Bray–Curtis dissimilarity was calculated between the microbial communities from each mangrove, followed by a principal coordinate analysis (PCoA). The first two principal coordinate (PC) axes explained a substantial proportion of the total variance (PC1 = 58.1%, PC2 = 15.7%). The resulting ordination plot revealed a clear segregation between the microbial compositions of the two mangroves, suggesting distinct community structures at each site (Fig. 2C).

**Fig. 3:**
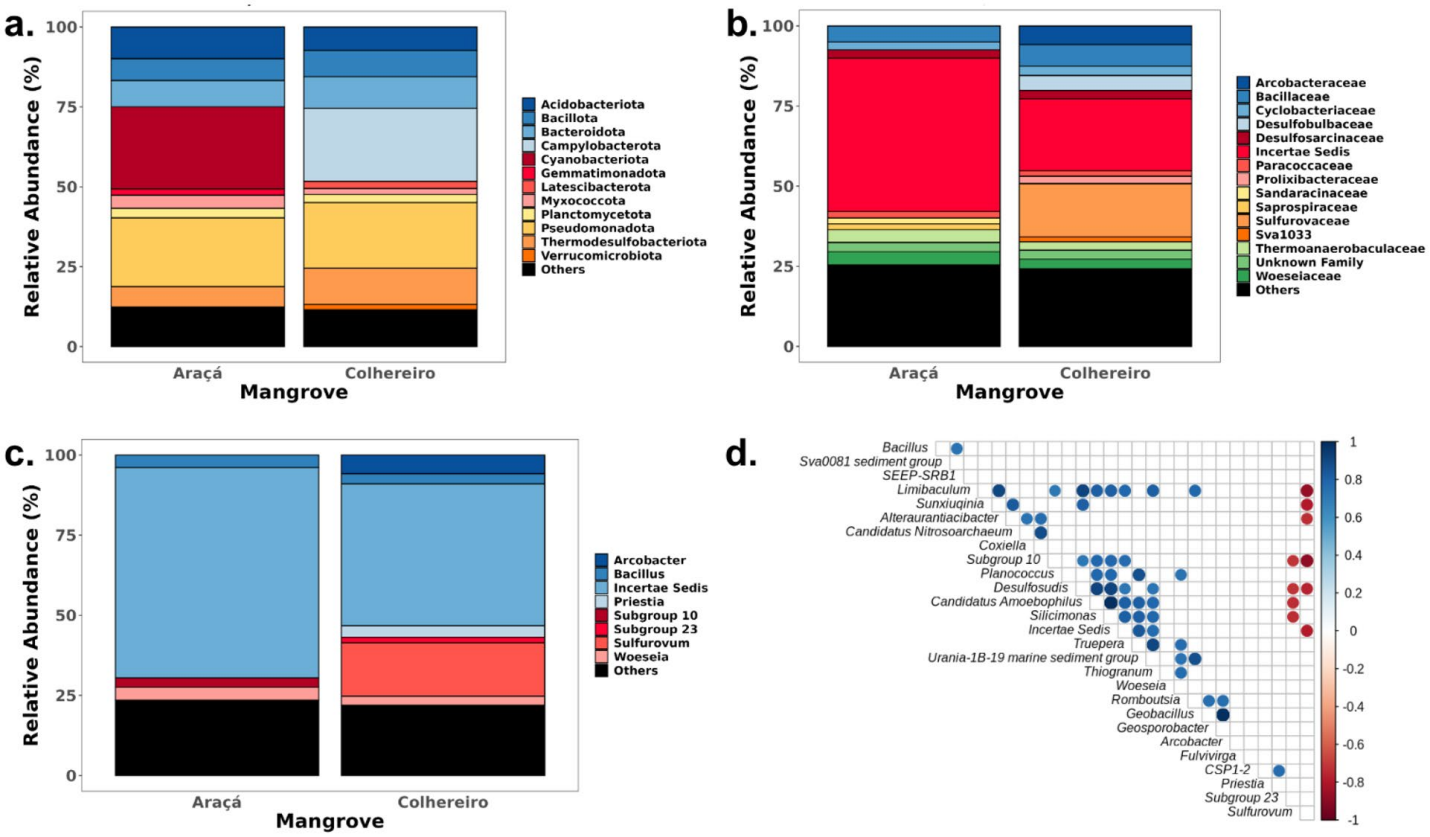
Taxonomic composition and microbial co-occurrence patterns in mangrove sediment microbiota. **(A–C)** Stacked bar plots showing the relative abundances of the most representative microbial taxa identified by 16S rRNA gene amplicon sequencing, displayed at three taxonomic levels: **(A)** phylum, **(B)** family, and **(C)** genus. Genera with relative abundances ≤ 1.5% in each sample were grouped into the category “Others” and shown in black. **(D)** Spearman correlation heatmap illustrating pairwise associations among identified genera in mangrove samples. Positive and negative correlations are depicted in shades of blue and red, respectively. Circle size represents the strength (absolute value) of the correlation coefficient, and color intensity is proportional to its magnitude. Only statistically significant correlations (*p* < 0.05) are shown; non-significant pairs appear as empty squares. For interpretability, genera are ordered identically on both axes to ease visual tracking of each pairwise relationship (Color figure online)

It is notable that the two mangrove sites exhibit distinct differences in the relative abundance of dominant microbial taxa (Fig. 3). At the phylum level, both Araçá and Colhereiro sediments were primarily composed by the phyla Thermodesulfobacteriota, Pseudomonadota, and Planctomycetota (Fig. 3A). While Araçá Bay exhibited significantly higher relative abundances (*p* = 0.028) of the phylum Cyanobacteriota (19.83% ± 17.16) compared to Colhereiro (1.34% ± 1.09), the latter presented a higher abundance (*p* = 0.028) of the phylum Campylobacterota (23.5% ± 8.43) compared to the former (1.2% ± 1). At the family level, Colhereiro harbored a greater abundance of sulfur-metabolizing bacteria, particularly members of the family Sulfurovaceae (*p* = 0.03), in contrast to Araçá. This was further supported by the significantly higher representation of the genus *Sulfurovum* in Colhereiro (*p* = 0.028). Additionally, the genus *Arcobacter* was more prevalent in Colhereiro, representing 5.65% ± 7.78 of all identified members in this community, whereas in Araçá it was only a minor member of the community (0.62 ± 0.96). Conversely, Araçá Bay was enriched with members of the genera *Bacillus* and *Woesia* compared to Colhereiro, although no statistical significance was identified. In both sites, a substantial proportion of the microbial community consisted of uncultured or poorly characterized taxa, including several lineages classified only to provisional groups (e.g., Subgroup 10 or *incertae sedis*), reflecting the complex and largely unexplored microbial diversity of mangrove sediments.

To infer co-occurrence patterns among the most prominent microbial members at each studied site, Spearman's rank correlation coefficients were calculated for all unique genera in each mangrove, with significant results plotted in a heat map (Fig. 3D). Only statistically significant correlations (*p* < 0.05) are displayed, with blue and red circles indicating positive and negative associations, respectively. This analysis revealed distinct clusters of co-occurring genera, including strong positive correlations among taxa such as the genera *Silicimonas*, *Planococcus*, and *Geobacillus*, as well as notable negative associations involving the genera *Sulfurovum* and *Desulfosudis*. These patterns suggest that specific microbial relationships may be conserved across mangrove environments, potentially reflecting shared ecological niches or competitive exclusion.

### Growth Characterization of Bacterial Cultures in Different Leachate Landfill Concentrations

**Fig. 4:**
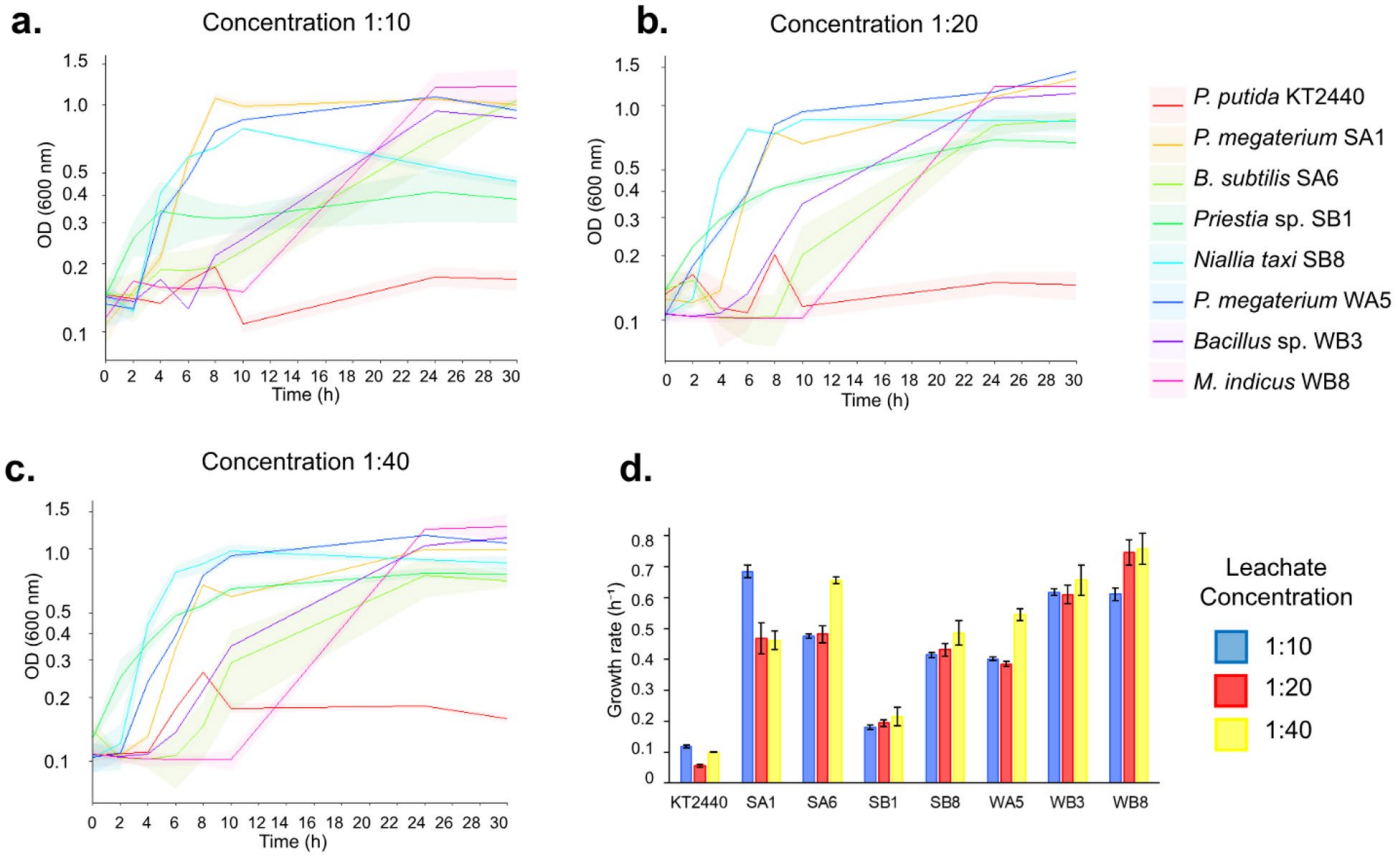
Growth curves in leachate at dilutions of 1:10 **(A)**, 1:20 **(B)**, and 1:40 **(C)** with different bacteria isolated from mangroves. The growth curves were constructed using three biological replicates. The graph represents the mean OD_600nm_ of three replicates and along with their standard deviation (SD). A factor of 3.0 should be applied to correct the optical path lengths for figures **A**, **B**, and **C**. (**D**) Growth rates were determined in the exponential phase for each concentration using the linear method represented by the mean ± SD

In terms of the cultivable share of the community, after a functional screening, seven bacteria presented higher growth rates, calculated on the OD_600nm_ measurements, when incubated for 30 h in landfill leachate as the sole nutrient source (Fig. 4). Among these, four bacterial cultures originated from sediment samples (SB1 and SB8 from Colhereiro; SA1 and SA6 from Araçá), while three were recovered from the overlying water collected at the sediment–water interface (WA5 from Araçá; WB3 and WB8 from Colhereiro). Detailed information on mangrove bacteria origin, sequencing data, and associated accession numbers is provided in Table S7. The taxonomic assignment of these mangrove bacteria is detailed in Sect. "Whole-genome sequencing, taxonomic affiliation, and genome-mining of promising bacterial cultures". These seven bacterial cultures were selected from 50 initially recovered cultivable isolates, based on their ability to reach higher final OD₆₀₀ values and/or exhibit sustained exponential growth compared to the remaining screened isolates.

When incubated at the 1/10 leachate dilution (Fig. 4A), differences in growth dynamics among bacterial cultures became evident. *Priestia megaterium* SA1 and WA5 displayed shorter lag phases (≈2 h) followed by rapid exponential growth, reaching stationary phase earlier than the other bacterial cultures. In contrast, *Metabacillus indicus* WB8 exhibited a prolonged lag phase (≈10 h) but achieved higher growth rates and final OD₆₀₀ values at later time points. At the 1/20 leachate dilution (Fig. 4B), *P. megaterium* WA5 and SA1, *M. indicus* WB8, and *Bacillus* sp. WB3 showed the highest growth rates, whereas at the 1/40 dilution (Fig. 4C), growth profiles converged across all bacterial cultures, with only minor differences in OD₆₀₀ trajectories. Across all leachate concentrations, *P.putida* KT2440 exhibited consistently lower growth rates and final OD₆₀₀ values compared to the mangrove-derived bacteria. Analysis of specific growth rates (Fig. 4D) confirmed that *P. megaterium* SA1 and WA5 performed best at the 1/10 dilution, while *M. indicus* WB8 achieved the highest growth rates at the 1/20 and 1/40 dilutions.

### Whole-Genome Sequencing, Taxonomic Affiliation, and Genome-Mining of Promising Bacterial Cultures

Considering the expressive growth of isolated bacteria from mangrove fragments in São Sebastião in urban leachate, whole-genome sequencing was employed to assess their taxonomic affiliation and allow for genome mining to discover additional possible biotechnology applications for these bacterial cultures. Considering all seven sequenced bacteria, 9.37 ± 0.53 million paired-end reads were generated per library (Table S4). De novo assemblies resulted in draft genomes ranging from 3.86 to 6.52 Mb, with GC contents between 37 and 44%. Genome quality assessment using CheckM2 indicated high completeness for all assemblies (99.67–100%), coupled with low contamination levels (0.00–2.15%), supporting their suitability for downstream comparative and functional genomic analyses. Assembly contiguity varied among bacterial cultures, with N50 values ranging from 147,213 to 1,929,202 bp and total contig numbers between 56 and 161. Predicted coding sequence counts ranged from 3,988 to 6,968 CDS per genome, consistent with genome size variation among the mangrove bacteria (Table S5, Figure S5). Based on the phylogenomic placement and digital DNA-DNA hybridization (dDDH) values, the bacterial cultures were assigned to *Priestia megaterium* SA1, *P. megaterium* WA5, *Bacillus subtilis* SA6, *Niallia taxi* SB8 and *Metabacillus indicus* WB8. Nonetheless, two representatives within the family Bacillaceae were identified as *Bacillus* sp. WB3 (*d4* = 63.4%) and *Priestia* sp. SB1 (*d4* = 68.6%) (Figures S6-S910 Tables S6 and S7). The former clustered with *Bacillus pumilus* with a bootstrap value of 75%, and the latter clustered with *Priestia aryabhattai* with an 88% bootstrap value. Thus, considering that both representatives presented dDDH values below the conventional 70% threshold used for species delineation and were placed in distinct clades with high bootstrap values, they may represent novel species within the genera *Bacillus* and *Priestia.*. Still, their classification as novel species requires additional phylogenomic analyses, as well as physiological and biochemical characterization, along with deposition in an international culture collection. These steps remain avenues for future investigation to conclusively determine their taxonomic status and explore their potential biotechnological applications. Complementary 16S rRNA gene-based phylogenetic analyses supported the genus-level placement of all bacterial cultures, with consistent clustering within their respective lineages (Figure S10).

**Fig. 5:**
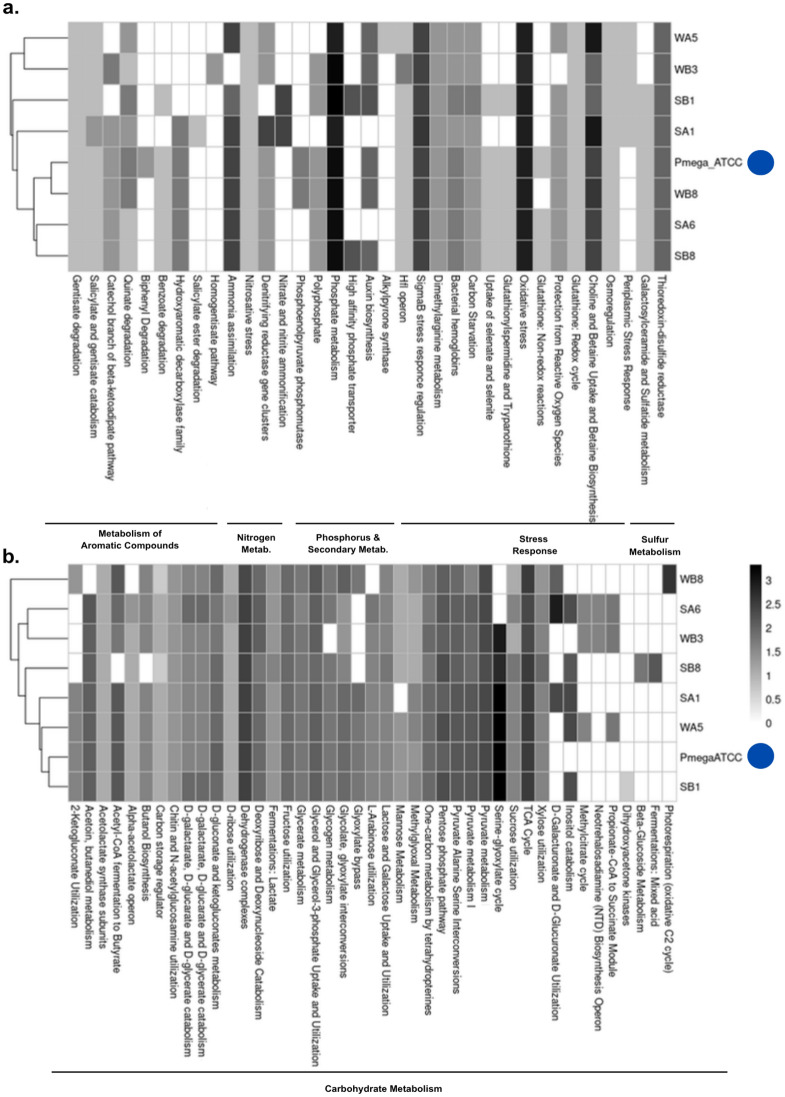
Genome mining of sequences putatively involved in **A.** traits of interest for bioremediation applications and in **B.** carbohydrate metabolism in isolated mangrove bacteria. The color scale represents the log-normalized feature counts for each bacteria. For comparison purposes, we included in the feature heat map the reference genome of *P. megaterium* ATCC 14581, highlighted by a blue circle (Color figure online)

These genomes were further screened for genes putatively involved in the adaptive growth in urban leachate, as well as finding additional features that could be harnessed when considering bioremediation applications (Fig. 5). When aligning the annotated genomes against the CARD database, only three bacterial cultures carried ARGs *(lsaB—P. megaterium* SA1; *BPU-1, cat86—Bacillus* sp. WB3; 12—*B. subtilis* SA6), all commonly reported in soil bacteria (Table S8). When performing the same workflow using VFDB and Plasmidfinder databases in the ABRICATE pipeline, no VGs or plasmids were identified in sequenced genomes. The annotation performed by RAST indicated the presence of several sequences involved in the metabolism of aromatic compounds, nitrogen, phosphorus, secondary, and sulfur metabolism in all sequenced bacteria (Fig. 5A). Nonetheless, the most prominent traits in all genomes derive from sequences involved in stress response, with an average number of ~ 35 distinct sequences/genome. Genomes from *B. subtilis* SA6 and *M. indicus* WB8 presented the highest (38) and lowest (30) copies of sequences involved in stress resistance, respectively. The sulfur metabolism was similar in all genomes, with thioredoxin-disulfide reductases corresponding to the most common subsystem for this trait. The same similarity among sequenced genomes was observed when comparing the phosphate metabolism and auxin biosynthesis, the only exception being *B. subtilis* SA6 and *Priestia* sp. SB1, which did not present any annotated sequences for auxin biosynthesis. In terms of nitrogen metabolism, the genomes presented an average number of 10 sequences, with most of them harboring denitrifying reductase gene clusters and ammonia assimilation sequences. Interestingly, *B. subtilis* SA6 presented the highest copies (19) of unique sequences involved in nitrogen metabolism. Overall, all mangrove bacteria sequenced harbored sequences involved in the metabolism of aromatic compounds, with an average number of ~ 8.6 sequence copies. *P. megaterium* SA1 and *B. subtilis* SA6 presented the highest genomic capability to metabolize aromatic compounds, including gentisate, biphenyl, and benzoate. Comparing these findings with the reference genome of *P. megaterium* ATCC 14581, the main differences observed among the mangrove-derived bacterial cultures and the reference genome involved (i) the metabolism of aromatic compounds (homogentisate pathway and salicylate degradation, present in SA1 and WB3, respectively), (ii) nitrogen (ammonification, present in SA1 and SB1), and (iii) phosphorus (phosphate transporter, present in SB1 and SB8). Moreover, the carbohydrate metabolism was also compared across sequenced mangrove bacteria (Fig. 5B). Most pathways related to carbohydrate metabolism were conserved in terms of both presence and gene copy number. However, some pathways were exclusive to specific sequenced bacteria, including: (i) dihydroxyacetone kinases, present only in *P. megaterium* SA1; (ii) beta-glucoside metabolism, exclusive to *Niallia taxi* SB8; and (iii) photorespiration, present only in *Metabacillus indicus* WB8. A complete list of all features identified during genome mining can be accessed in the supplementary files. Notably, these pathways identified in specific bacteria were not annotated in the *P. megaterium* ATCC 14581 reference genome, highlighting the metabolic versatility of these bacteria and their potential for biotechnological applications in nutrient cycling and bioremediation.

## Discussion

Mangroves are remarkable ecosystems that display diverse and complex microbial communities, being reservoirs of bacteria with significant biotechnological potential in distinct areas [53]. These coastal wetlands are crucial for ecological functions and services; however, they face rapid decline globally due to anthropogenic pressures [54]. Understanding the microbial diversity within these ecosystems is not only vital for their conservation but also for harnessing the unique capabilities of these microorganisms for biotechnological applications. In the present study, we characterized the microbiota of Araçá and Colhereiro mangroves, located on the northern São Paulo coast. São Sebastião, the municipality where these mangroves are located, contains five main mangrove fragments, all of which are in bad conservation status [8]. Even those within permanently protected areas, such as Araçá, are significantly affected by urban expansion, pollution, and harbor activities [6]. The chemical analysis of the mangroves’ sediments demonstrates that Colhereiro presents higher levels of macronutrients (i.e., P, Ca^2^⁺, Mg^2^⁺, K⁺) and organic matter compared to Araçá (Table S1), though both sites suffer from contamination from the portuary activity in the vicinity (Fig. 1) [55]. The observed differences in sediment chemical parameters between the two mangrove sites likely reflect the distinct anthropogenic pressures shaping each environment. Colhereiro, a small, secondary mangrove fragment that developed following landscape disturbances, is heavily affected by urban-derived pollution [8]. Its proximity to the São Sebastião ferry terminal exposes it to intense pedestrian traffic, illegal waste disposal, and minimal enforcement of environmental regulations [17]. These stressors likely contribute to elevated levels of organic matter, nutrients, or contaminants observed in the site’s sediment profile. In contrast, Araçá Bay, although ecologically richer and more extensive, has experienced severe fragmentation and hydrodynamic alteration due to the expansion of the São Sebastião Port and dredging operations [6]. These infrastructural changes have disrupted the natural tidal regime and intensified sedimentation within the bay [6]. Therefore, while Colhereiro’s environmental degradation is primarily linked to localized urban inputs and chronic pollution, Araçá’s biogeochemical shifts may be more closely associated with habitat fragmentation and altered hydrological dynamics [8]. This explains not only the differences in the analyzed chemical parameters but also the clear clustering of samples’ microbial composition according to their corresponding mangrove (Fig. 2C), which highlights previous reports discussing the use of microbiota to infer soil health in different environments [56, 57]. In this study, we found that Araçá’s bacterial community exhibited lower alpha diversity values (both Shannon and Simpson indices) than Colhereiro mangrove (Fig. 2). This seemingly paradoxical result may reflect the lower evenness and reduced presence of rare taxa in Araçá’s microbiota, which could be linked to habitat fragmentation and increased sedimentation that limit niche heterogeneity and dispersal [58]. Nevertheless, its community structure, in terms of taxonomic richness and composition, was consistent with reports from less impacted mangrove ecosystems on the Brazilian coast [56, 57], underscoring a potential resilience of its microbiota despite physical alterations from dredging and port expansion [58–61]. In contrast, the Colhereiro mangrove exhibited higher alpha diversity indices, which may initially suggest greater microbial richness and evenness. However, such increases in diversity are often reported in polluted or disturbed environments, where stress-induced niche availability can support opportunistic taxa [62–64]. Similar patterns have been reported in other disturbed tropical ecosystems, where shifts in soil physicochemical properties driven by land-use change strongly restructure microbial community composition while partially preserving functional potential during ecosystem recovery [65].

Sulfur-metabolizing bacteria are considered part of the core mangrove microbiome, playing essential roles in anaerobic respiration and sulfur cycling. Importantly, several of these sulfur-cycling taxa belong to strictly anaerobic lineages, including members of the phylum Thermodesulfobacteriota, which emerged as one of the main drivers of community structure in sediment samples. This highlights the central role of anaerobic metabolism in mangrove sediments, where reduced conditions favor sulphate reduction and other anaerobic processes critical for organic matter turnover and contaminant transformation [66, 67]. However, their enrichment has been consistently associated with anthropogenic stressors, such as urban runoff, and sewage contamination [68–71]. Colhereiro microbial communities were notably dominated by sulfur-metabolizing genera such as the genus *Sulfurovum* (Fig. 3C), taxa previously linked to hydrocarbon degradation, heavy metal resistance, and bioremediation capacities [72–75]. This dominance, coupled with a marked reduction in cyanobacteria (Fig. 3A, 3B), aligns with previous reports of polluted urban mangrove environments in Brazil [76–78]. Cyanobacteria have been shown to play key roles in carbon and nitrogen fixation, phosphorus storage, and bioremediation in mangrove ecosystems, and their depletion in polluted sites may therefore compromise essential ecosystem functions and restoration potential [79]. Moreover, we identified a high relative abundance of the genus *Arcobacter* within the Colhereiro mangrove (Fig. 3C), a genus that includes several emerging opportunistic and zoonotic pathogens [80]. These bacteria are often associated with fecal contamination and are frequently detected in wastewater treatment plant effluents and urban-impacted aquatic environments [81, 82]. These characteristics underscore their capability to thrive in polluted aquatic environments and pose potential public health risks [83]. The prominence of the genus *Arcobacter* in Colhereiro sediments suggests the presence of niche opening driven by chronic exposure to sewage and urban runoff. This opportunistic proliferation aligns with patterns seen in other coastal ecosystems impacted by aquaculture and urban waste, where the genus *Arcobacter* emerges as a marker of environmental degradation and potential human health hazard [81]. Consequently, the genus *Arcobacter* spp. may serve as a sensitive, pollution-linked bioindicator for degraded mangrove systems, warranting further investigation regarding its correlation with anthropogenic inputs and its role in microbial community reorganization.

Urban mangrove fragments are frequently contaminated by leachate from human waste, highlighting the crucial role of these ecosystems in buffering contaminants and preventing broader aquatic pollution [84, 85]. Leachate, a byproduct of the waste decomposition process in landfills, contains a complex mixture of organic and inorganic compounds, including toxic metals, ammonia, volatile organic compounds (VOCs), and various other pollutants [86]. Its toxicity presents a significant challenge for biological treatment due to the presence of high concentrations of salts, heavy metals, and recalcitrant compounds [87]. While leachate’s composition may impair bacterial communities [73], several bacteria are commonly used in landfill leachate treatment, such as nitrifying bacteria that play a crucial role in the removal of ammoniacal nitrogen through nitrification processes [70, 88]. Other studies have demonstrated the efficacy of using microbial consortia for enhanced leachate biodegradation [89] and mangrove in situ decontamination [90, 91]. Indigenous microbial consortia have proven effective for in situ bioremediation of mangroves polluted by urban waste, as native microbial communities are better adapted to the unique challenges of these ecosystems, outperforming exogenous bacteria and physical or chemical remediation methods [92, 93]. This study highlights the differential growth responses of mangrove bacteria in landfill leachate at various dilutions, with the potential of microbial consortia from these bacterial cultures yet to be explored for enhanced bioremediation. Key genera such as *Bacillus* and *Priestia* were abundant and strongly correlated with the mangrove microbiota (Fig. 3), while the genus *Metabacillus*, though less common (~ 0.15% in Araçá Bay), is noted for its significant bioremediation potential [94]. Although elevated leachate concentrations typically suppress bacterial growth, *P. megaterium* SA1, *Bacillus* sp. WB3 and *M. indicus* WB8 demonstrated notable adaptability and significant growth under the highest concentration tested (Fig. 4). This phenotype is likely attributable to increased nutrient availability and underscores their potential application in leachate bioremediation efforts [95], as previously reported in studies that explored the use of mangrove *Bacillus* species as effective bioremediators in distinct contaminated environments [96–98]. Genome mining of the most promising bacterial cultures further supports their observed phenotype and their potential for bioremediation applications. Genomic analysis revealed genes involved in the metabolism of aromatic compounds, nitrogen, phosphorus, and sulfur cycling, along with a notable presence of stress response genes, aligning with the leachate-degradation phenotype observed in the mangrove bacteria (Fig. 5). These results are consistent with previous studies demonstrating the resilience of mangrove bacteria to environmental stressors, emphasizing their potential for environmental cleanup and industrial waste treatment [99, 100]. Notably, *B. subtilis* SA6 and *P. megaterium* SA1 exhibited unique metabolic traits, particularly in ammonia assimilation and aromatic compound metabolism, which are valuable for managing common soil pollutants [101, 102]. Furthermore, sequences related to phosphate metabolism and auxin biosynthesis were identified in *N. taxi* SB8, suggesting it could enhance soil fertility through improved nutrient cycling [103]. Comparative analysis of carbohydrate metabolic pathways revealed distinctive traits among sequenced bacteria: dihydroxyacetone kinases were found exclusively in *P. megaterium* SA1, beta-glucoside metabolism was unique to *N. taxi* SB8, and photorespiration was observed only in *M. indicus* WB8. These specialized metabolic capabilities imply that each bacteria may fulfill distinct roles in nutrient cycling within mangrove ecosystems [104, 105]. For instance, dihydroxyacetone kinases have been linked to carbon storage and utilization, which could provide *P. megaterium* SA1 with a competitive advantage in nutrient-limited environments like mangrove sediments [100]. Additionally, beta-glucoside metabolism in *N. taxi* SB8 could enhance the breakdown of cellulose and other plant-derived carbohydrates, contributing to the organic matter cycle [106]. Furthermore, the absence of clinically relevant antibiotic resistance genes (ARGs) and virulence genes (VGs) in these bacterial cultures underscores their safety and enhances their suitability for various biotechnological applications (Table S8).

Despite providing valuable insights into the microbial diversity and bioremediation potential of urban mangrove sediments, our study has several limitations that must be addressed to achieve a more holistic understanding of Brazilian mangrove microbiomes. First, the analysis was based on a single time-point sampling, limiting our ability to capture temporal variability in microbial communities. Longitudinal studies are needed to monitor seasonal or anthropogenic changes over time. Second, microbial interactions were not assessed through co-occurrence or network analyses, which could reveal important ecological relationships and community dynamics within each site. Third, although some bacterial cultures exhibited promising responses to leachate exposure, the effectiveness of microbial consortia remains to be tested, particularly under environmentally relevant conditions. The role of anaerobic microorganisms—especially those active in deeper sediment layers—also warrants further exploration, given their importance in complete contaminant breakdown. Finally, functional characterization of bacterial cultures through experimental validation of degradation pathways and field-scale testing is necessary to confirm their potential for applied bioremediation. These limitations emphasize the need for integrative, multidisciplinary approaches combining genomics, functional assays, and ecological modelling to fully unravel the microbial complexity and restoration potential of degraded mangrove systems. Despite these limitations, this study provides, to our knowledge, the first integrated taxonomic, functional, and cultivation-based characterization of the microbiota from the Araçá and Colhereiro mangroves. As these systems have not been previously investigated at this resolution, our results constitute an important baseline snapshot of their microbial communities. In the event of future restoration initiatives or further environmental degradation, this dataset offers a valuable reference point for assessing microbiome shifts and ecosystem recovery trajectories.

## Conclusions

Mangroves are essential coastal wetland ecosystems that support diverse and complex microbial communities, which play a crucial role in maintaining ecological functions and services. Despite their ecological and economic significance, these ecosystems are undergoing rapid degradation worldwide due to increasing anthropogenic pressures. This study highlights the impact of environmental degradation on the taxonomic and functional profiles of sediment microbiota from two urban mangrove fragments on the northern coast of São Paulo. While the Araçá mangrove exhibited features of a moderately conserved ecosystem, Colhereiro’s microbiota reflected hallmarks of chronic urban pollution, including the proliferation of sulfur-metabolizing bacteria and opportunistic taxa such as the genus *Arcobacter*. Despite these contrasting microbial signatures, both sites harbored indigenous bacteria with potential for bioremediation, particularly under landfill leachate stress. These insights advance our understanding of mangrove microbiomes and emphasize their relevance for ecosystem monitoring and restoration. As an exploratory study, this work is limited by its single time-point sampling and the need for functional validation of candidate degraders. Addressing these gaps will be crucial for future investigations aiming to assess the resilience and restoration capacity of Brazil’s increasingly fragmented and vulnerable mangrove ecosystems. Importantly, this work provides the first integrated snapshot of the microbial diversity and functional potential of the Araçá and Colhereiro mangroves. As these systems had not been previously characterized at this resolution, our findings establish a baseline reference for future monitoring, restoration efforts, or assessments of further environmental disturbance.

## Supplementary Information

Below is the link to the electronic supplementary material.

Supplementary file1 (DOCX 1228 KB)

Supplementary file2 (TXT 208 KB)

Supplementary file3 (TXT 82 KB)

## Data Availability

Raw amplicon and whole-genome sequencing data have been deposited in the Sequence Read Archive (SRA) and genome repositories hosted by NCBI under the BioProject accession number PRJNA1042624. Individual SRA run accession numbers (SRR identifiers) for each sample are provided in Table S2 (amplicon sequencing data) and Table S7 (genome sequencing data). Additional features identified during genome mining are available in the supplementary file RAST_genome-mining.zip.

## Code availability

All codes used during the development of this project can be accessed at GitHub (https://github.com/joaoordine/mangrove_microbiota).
