# Supplementary material for: Taxonomic and Functional Assessment of Microbial Communities in Urban Runoff-Contaminated Mangrove Sediments
Source: Curr Microbiol. 2026 Apr 16;83(6):303. doi: 10.1007/s00284-026-04910-5 (PMC13086700; doi:10.1007/s00284-026-04910-5)
Supplement: Supplementary file 1 — Supplementary file1 (DOCX 1228 KB) [file 284_2026_4910_MOESM1_ESM.docx]

**Taxonomic and Functional Assessment of Microbial Communities in Urban Runoff-Contaminated Mangrove Sediments**

João Vitor Wagner Ordine^1^, Franciene Rabiço Oliveira^2^, Jonatã Bortolucci³, Lívia Soares Zaramela^1^, and María-Eugenia Guazzaroni^2^.

^1^ Department of Biochemistry, Ribeirão Preto Medical School, University of São Paulo, Ribeirão Preto, SP, Brazil; [joaoordine@usp.br](mailto:joaoordine@usp.br) - <https://orcid.org/0000-0002-2370-8971> (J.V.W.O.); [zaramela@fmrp.usp.br](mailto:zaramela@fmrp.usp.br) - https://orcid.org/0000-0002-1065-3799 (L.S.Z.);

^2^ Department of Biology, Faculty of Philosophy, Sciences and Letters of Ribeirão Preto, University of São Paulo, Ribeirão Preto, SP, Brazil; [meguazzaroni@ffclrp.usp.br](mailto:meguazzaroni@ffclrp.usp.br) - <https://orcid.org/0000-0002-4657-3731> (M-E.G.); [franciene.oliveira@usp.br](mailto:franciene.oliveira@usp.br) - <https://orcid.org/0000-0003-4382-9823> (F.R.O.);

^3^ Department of Chemistry, Faculty of Philosophy, Sciences and Letters of Ribeirão Preto, University of São Paulo, Ribeirão Preto 14049-900, Brazil; [jonata.bortolucci@usp.br](mailto:jonata.bortolucci@usp.br) - <https://orcid.org/0000-0001-6092-844X> (J.B.).

*Correspondence to:

María-Eugenia Guazzaroni, meguazzaroni@ffclrp.usp.br

Faculdade de Filosofia, Ciências e Letras de Ribeirão Preto, Universidade de São Paulo.

Av. Bandeirantes, 3.900. CEP: 14049-901, Ribeirão Preto, São Paulo, Brazil. Tel +55 (16) 3315 3680

**Supplementary Materials**

**Supplementary Methodology**

*Type Strain Genome Server (TYGS) whole genome-based taxonomic analysis*

Information on nomenclature, synonymy, and associated taxonomic literature was provided by the List of Prokaryotic names with Standing in Nomenclature (LPSN, available at [https://lpsn.dsmz.de](https://lpsn.dsmz.de/),accessed on 2024-03-07). Determination of the closest type strain genomes was done in two complementary ways: First, all submitted genomes were compared against all type strain genomes available in the TYGS database via the MASH algorithm, a fast approximation of intergenomic relatedness [51], and the ten type strains with the smallest MASH distances were chosen for each submitted genome. Second, an additional set of ten closely related type strains was determined via the 16S rDNA gene sequences. These were extracted from the submitted genomes using RNAmmer [52]. Each sequence was subsequently BLASTed against the 16S rDNA gene sequence TYGS database [53]. This was used as a proxy to find the best 50 matching type strains - according to the bitscore - for each submitted genome and to subsequently calculate precise distances using the Genome BLAST Distance Phylogeny approach (GBDP) under the algorithm 'coverage' and distance formula d5 [54]. These distances were finally used to determine each submitted genome's ten closest type strain genomes. For the phylogenomic inference, all pairwise comparisons among the set of genomes were conducted using GBDP, and accurate intergenomic distances were inferred under the algorithm 'trimming' and distance formula d5. Then, 100 distance replicates were calculated for each submitted genome to assess the reliability of the inferred branches. Digital DDH values and confidence intervals were calculated using the recommended settings of the GGDC v4.0. The resulting intergenomic distances were used to infer a balanced minimum evolution tree with branch support via FASTME v2.1.6.1, including SPR postprocessing [55]. Branch support was inferred from 100 pseudo-bootstrap replicates each. The trees were rooted at the midpoint and visualized with PhyD3 [56,57]. The type-based species clustering was done using a 70% dDDH (digital DNA–DNA hybridization) radius around each of the 16 type strains as previously described. The isolate was considered to represent a determined species when the corresponding d4 formula and phylogenetic branch support values were above 70%.


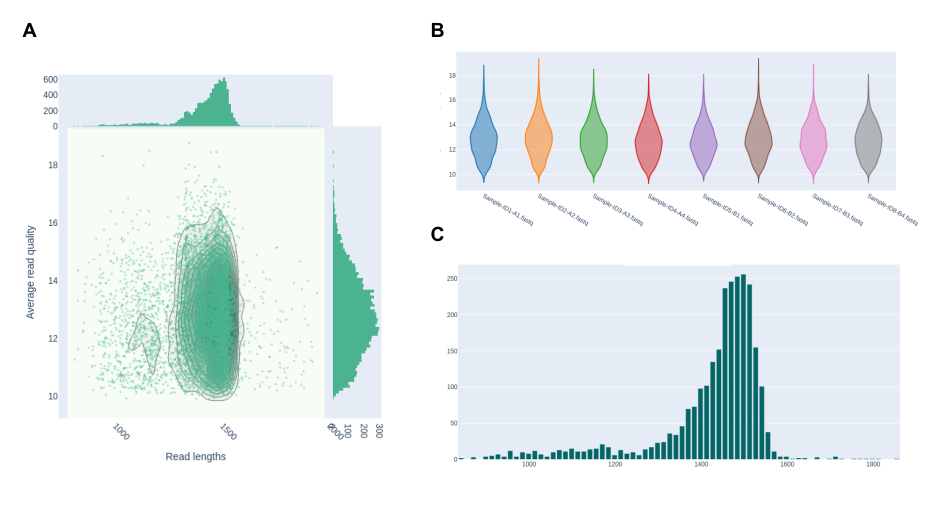
**Figure S1.** Quality control of whole community raw amplicon-reads before filtering and trimming steps.


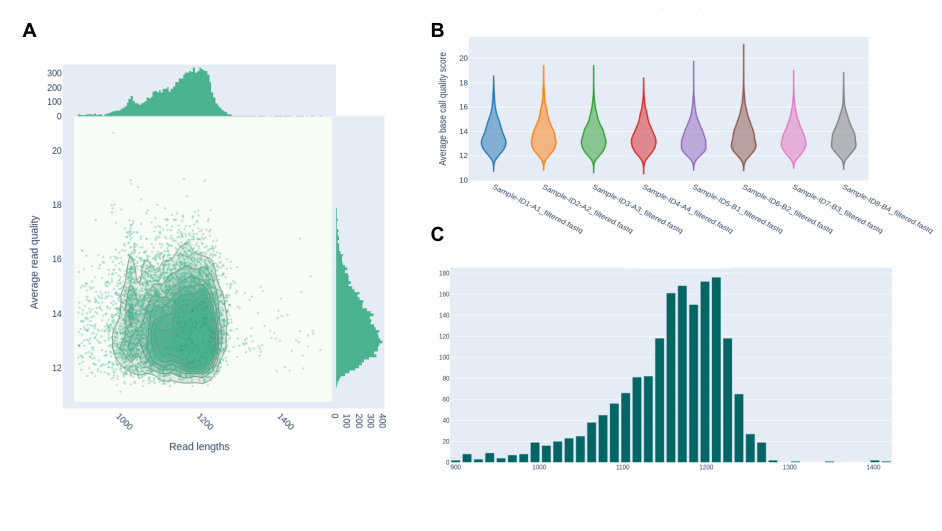
**Figure S2.** Quality control of whole community high-quality amplicon-reads after filtering and trimming steps.


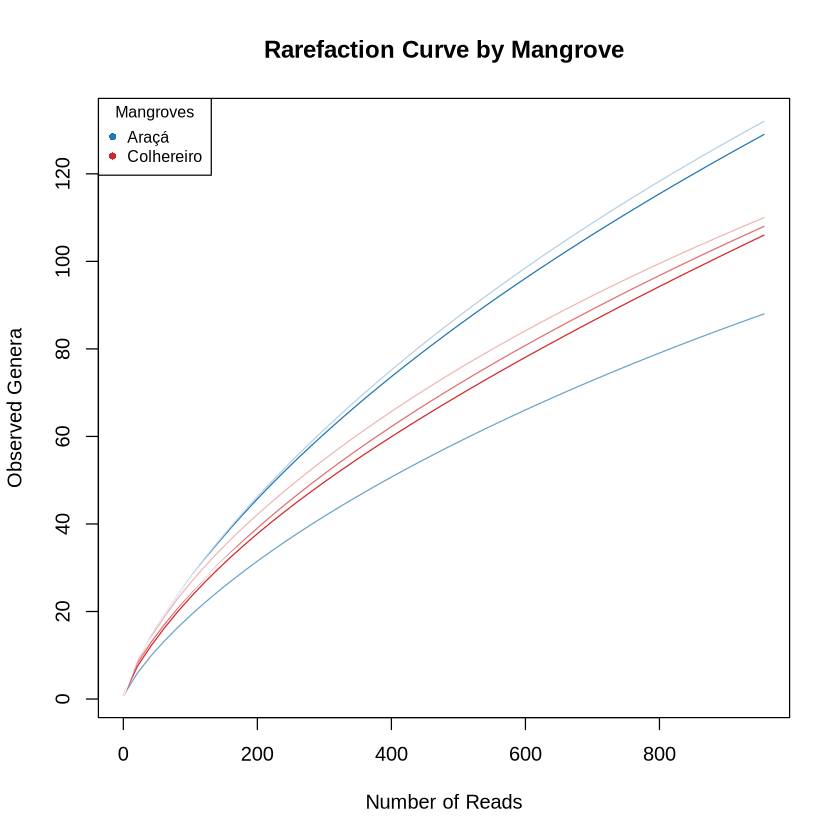


**Figure S3.** Collectors’ curves depicting the average number of genera detected per sample according to the sample size.


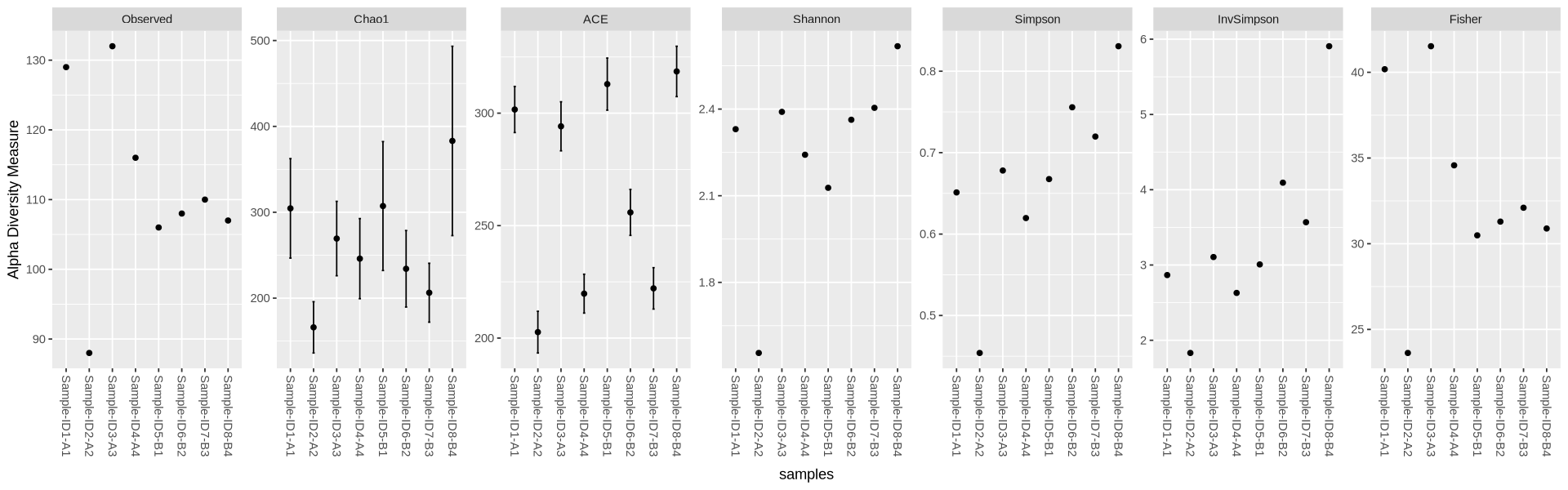
**Figure S4.** Scatter plots representing the calculated alpha diversity metrics for each mangrove replica.


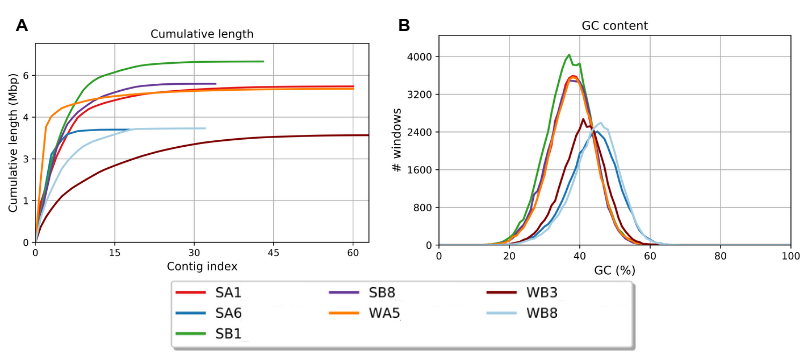


**Figure S5.** Summary of quality parameters of assembled genomes plotted by QUAST. **A.** Rarefaction curves depicting the cumulative length of the sequenced genomes with the contig increment. **B.** GC content observed in each sequenced genome.


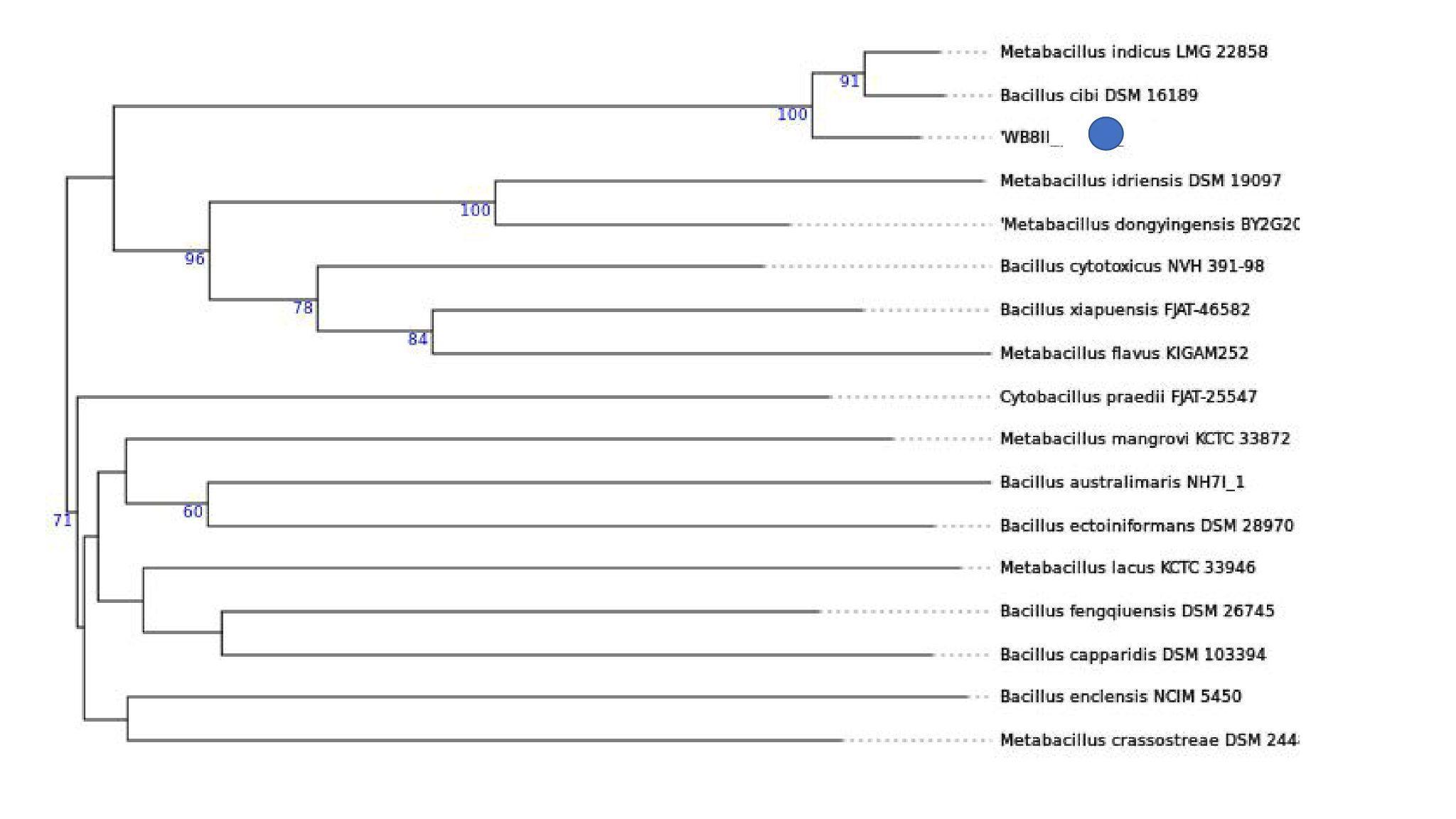


**Figure S6.** Tree inferred with FastME 2.1.6.1 from GBDP distances calculated from genome sequences. The branch lengths are scaled in terms of the GBDP distance formula *d5*. The numbers above branches are GBDP pseudo-bootstrap support values > 60 % from 100 replications, with an average branch support of 59.1 %. The tree was rooted at the midpoint.


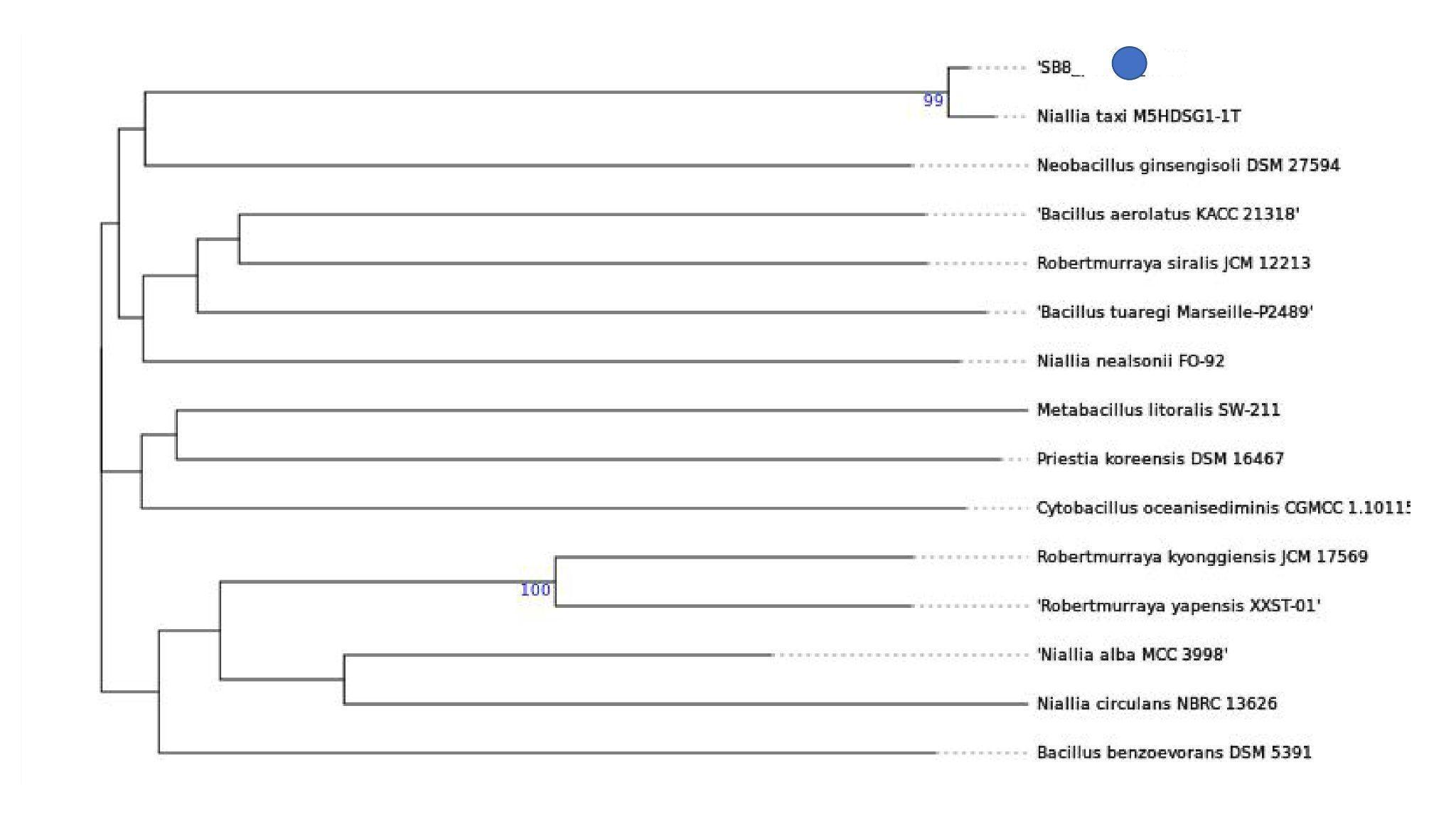


**Figure S7.** Tree inferred with FastME 2.1.6.1 from GBDP distances calculated from genome sequences. The branch lengths are scaled in terms of GBDP distance formula d5. The numbers above branches are GBDP pseudo-bootstrap support values > 60 % from 100 replications, with an average branch support of 41.9 %. The tree was rooted at the midpoint.


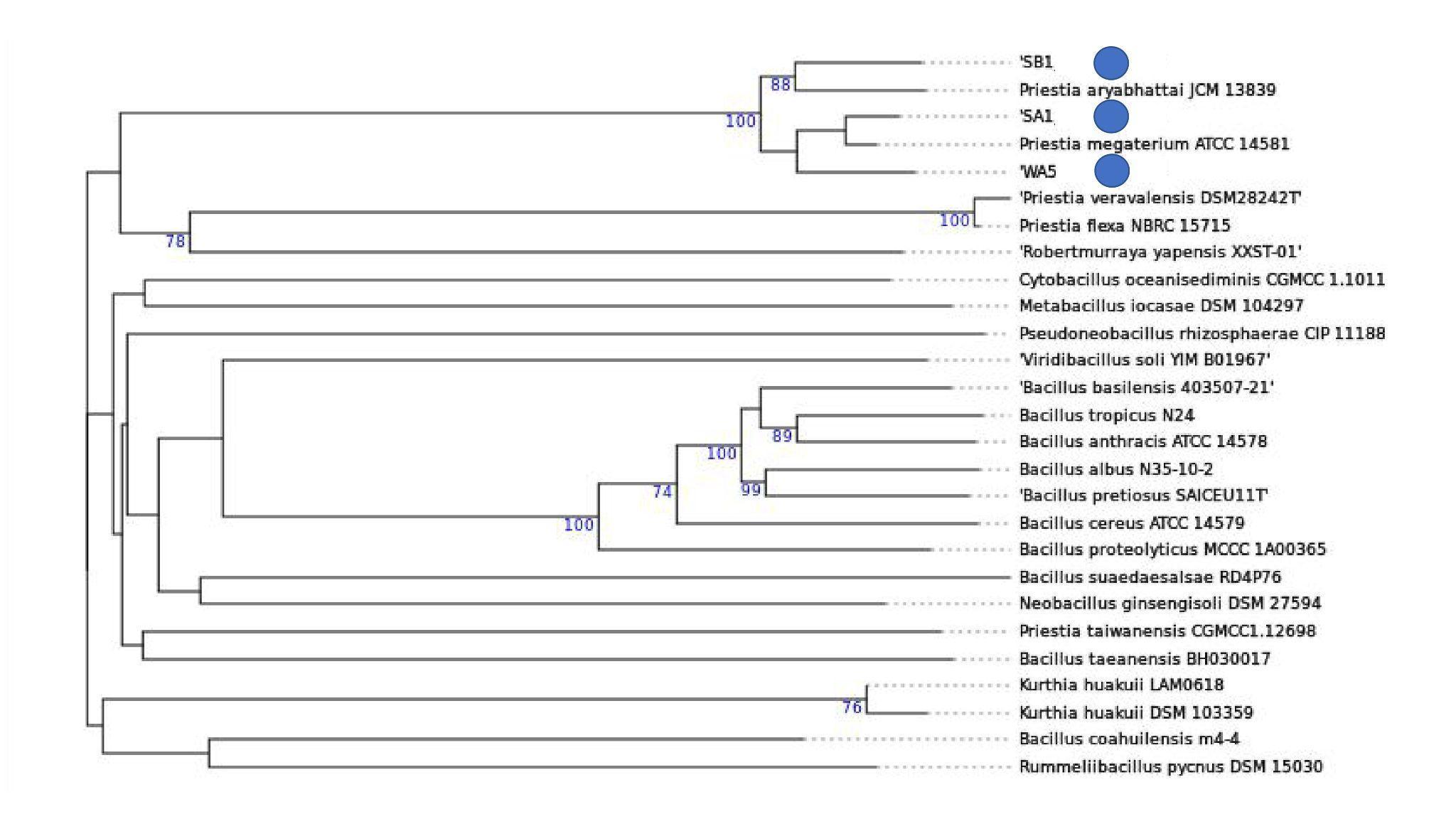


**Figure S8.** Tree inferred with FastME 2.1.6.1 from GBDP distances calculated from genome sequences. The branch lengths are scaled in terms of GBDP distance formula d5. The numbers above branches are GBDP pseudo-bootstrap support values > 60 % from 100 replications, with an average branch support of 51.6 %. The tree was rooted at the midpoint.


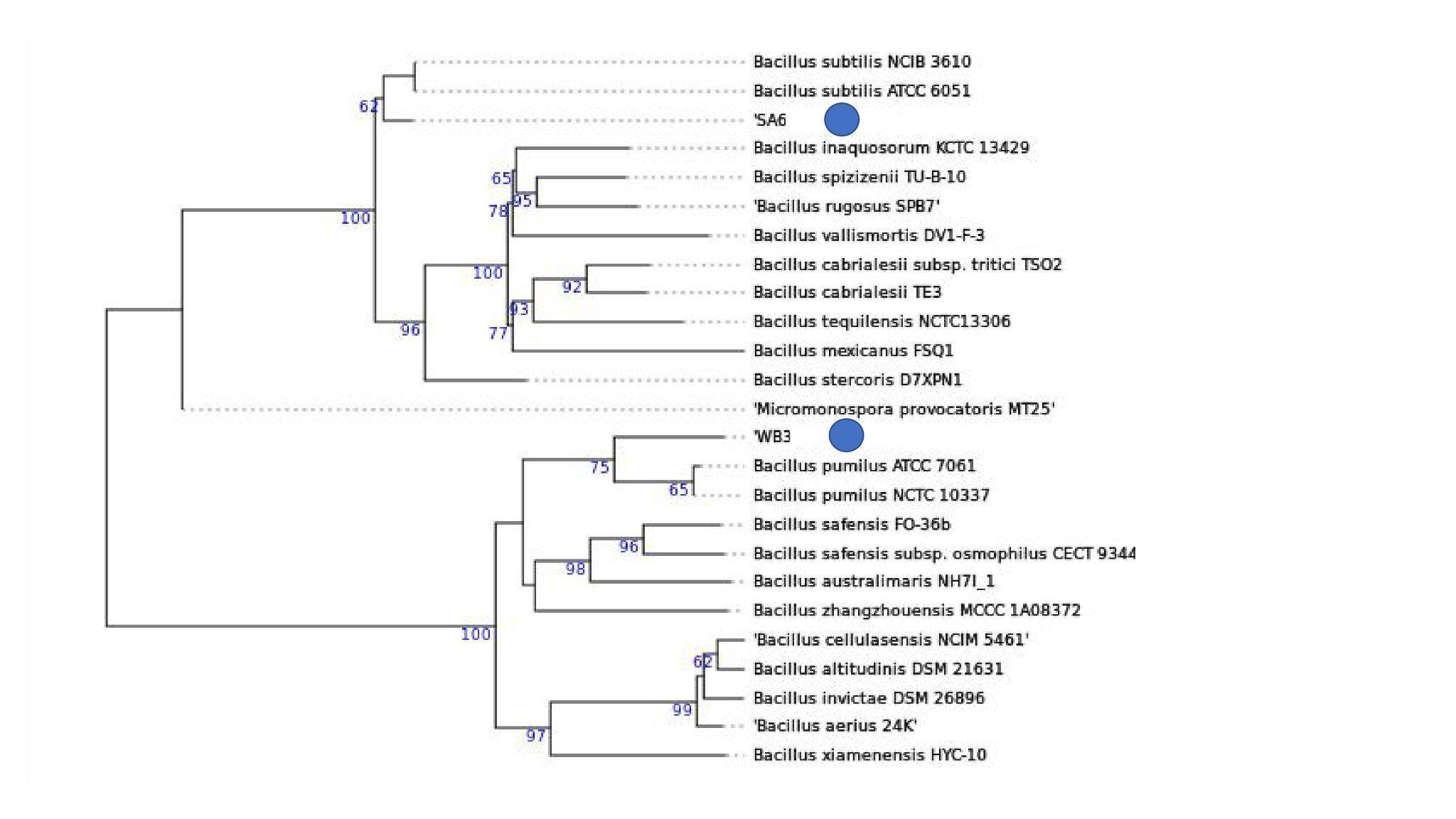


**Figure S9.** Tree inferred with FastME 2.1.6.1 from GBDP distances calculated from genome sequences. The branch lengths are scaled in terms of GBDP distance formula d5. The numbers above branches are GBDP pseudo-bootstrap support values > 60 % from 100 replications, with an average branch support of 77.8 %. The tree was rooted at the midpoint.


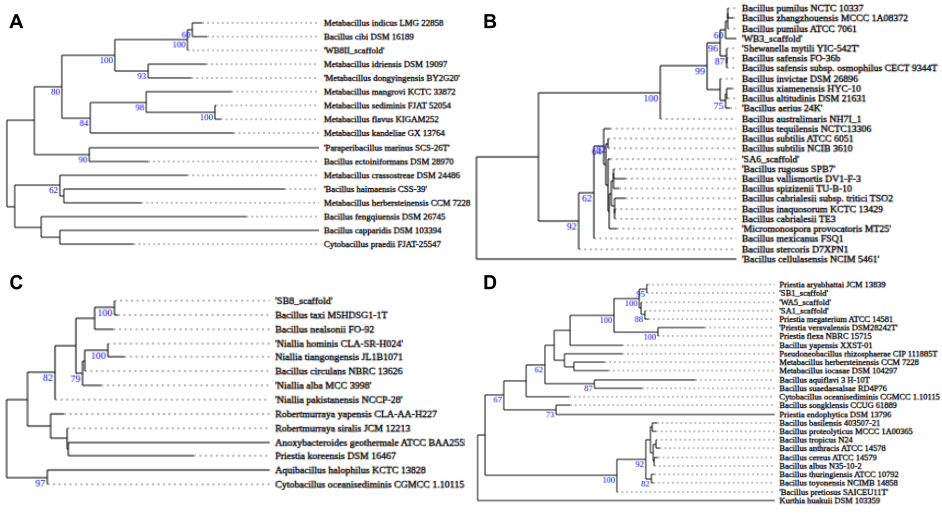
**Figure S10.** Trees inferred with FastME 2.1.6.1 from GBDP distances calculated from 16S rDNA gene sequences. The branch lengths are scaled in terms of GBDP distance formula *d5*. The numbers above branches are GBDP pseudo-bootstrap support values > 60 % from 100 replications. Trees correspond to the following isolates: **A.** WB8, with an average branch support of 73.9%. **B.** SA6 and WB3, with an average branch support of 53.9%. **C.** SB8, with an average branch support of 65.3%. **D.** SA1, WA5, SB1, with an average branch support of 65.4%. The trees were rooted at the midpoint.

**Table S1.** Summary of analyzed chemical parameters in the studied mangroves. Expression units: pH - CaCl₂; total organic matter (TOM) - g/dm³; phosphate (PO4³**⁻**) - mg/dm³; potassium (K**⁺**), calcium (**Ca²⁺**), magnesium (Mg²⁺), aluminum (**Al³⁻**), potential acidity (**H⁺+Al³⁺**), sum of exchangeable bases (SB) and cation exchange capacity (CEC) - mmolc/dm³; base saturation (V%); aluminum saturation (m%).

| **Mangrove**  **Samples** | **pH** | **TOM** | **PO4³⁻** | **Ca²⁺** | **Al³⁻** | **Mg²⁺** | **K⁺** | **H⁺+Al³⁺** | **SB** | **CEC** | **V%** | **m%** |
| --- | --- | --- | --- | --- | --- | --- | --- | --- | --- | --- | --- | --- |
| Araçá Bay  (A1-A4) | 7.3 | 10 | 24 | 58 | > 1 | 56 | 12.6 | 9 | 126.6 | 135 | 94 | 0 |
| Colhereiro  (B1-B4) | 7.8 | 47 | 33 | 122 | > 1 | 133 | 26.4 | 9 | 281.4 | 289 | 97 | 0 |

**Table S2.** Data availability and amplicon-sequenced reads count before and after filtering steps.

| **BioSample Accession** | **Sample** | **BioProject** | **SRA Accession** | **Raw Reads** | **Reads After Filtering** | **Reads > Q10** | **Reads > Q15** |
| --- | --- | --- | --- | --- | --- | --- | --- |
| SAMN38314155 | A1 | PRJNA  1042624 | SRR26872268 | 1,742 | 1,134 | 100% | 13% |
| SAMN38314156 | A2 |  | SRR26872267 | 4,344 | 2,971 | 100% | 15% |
| SAMN38314157 | A3 |  | SRR26872266 | 2,890 | 1,882 | 100% | 12% |
| SAMN38314158 | A4 |  | SRR26872265 | 1,628 | 965 | 100% | 12% |
| SAMN38314159 | B1 |  | SRR26872264 | 2,689 | 1,686 | 100% | 9,5% |
| SAMN38314160 | B2 |  | SRR26872263 | 3,540 | 2,375 | 100% | 15% |
| SAMN38314161 | B3 |  | SRR26872262 | 1,873 | 1,231 | 100% | 11% |
| SAMN38314162 | B4 |  | SRR26872261 | 2,669 | 1,693 | 100% | 11% |

**Table S3.** Summary of calculated alpha diversity metrics for each mangrove replica.

| **Sample** | **Mangrove** | **Chao1** | **ACE** | **Shannon** | **Simpson** | **Fisher** |
| --- | --- | --- | --- | --- | --- | --- |
| A1 | Araçá | 203 | 183.7297 | 3.02753 | 0.84882 | 39.99565266 |
| A2 |  | 142 | 144.6261 | 2.15240 | 0.66415 | 43.57024894 |
| A3 |  | 127.8 | 143.4170 | 3.06974 | 0.85306 | 54.52074153 |
| A4 |  | 260.5 | 197.3267 | 2.95560 | 0.80761 | 60.25205709 |
| B1 | Colhereiro | 147.7 | 152.0565 | 3.08360 | 0.89433 | 18.36332463 |
| B2 |  | 222.4 | 182.3593 | 2.99823 | 0.88100 | 7.886256237 |
| B3 |  | 149 | 160.7650 | 3.23734 | 0.89894 | 15.621098 |
| B4 |  | 147.1 | 158.4179 | 3.06664 | 0.90374 | 16.86174162 |

**Table S4.** Whole-genome sequencing quality parameters of selected mangrove isolates.

| **Sample** | **Sequenced**  **Raw Reads** | **Effectiveness**  **(%)** | **Error**  **(%)** | **Q20**  **(%)** | **Q30**  **(%)** | **GC**  **(%)** |
| --- | --- | --- | --- | --- | --- | --- |
| SB1 | 8911574 | 99.71 | 0.03 | 97.09 | 91.74 | 36.99 |
| SB8 | 8412248 | 99.76 | 0.03 | 97.37 | 92.34 | 37.44 |
| SA1 | 9850320 | 99.79 | 0.03 | 97.21 | 92.02 | 37.9 |
| SA6 | 9566068 | 99.74 | 0.03 | 97.38 | 92.53 | 43.56 |
| WA5 | 9385934 | 99.76 | 0.03 | 97.17 | 91.9 | 37.91 |
| WB3 | 9574998 | 99.02 | 0.03 | 97.39 | 92.43 | 38.09 |
| WB8 | 9910674 | 99.75 | 0.03 | 97.16 | 92.03 | 44.25 |

**Table S5.** Summary quality metrics of assembled bacterial genomes provided by CheckM2.

| Sample | Genome size (bp) | GC content (%) | Completeness (%) | Contamination (%) | N50  (bp) | Total contigs | Total CDS |
| --- | --- | --- | --- | --- | --- | --- | --- |
| SA1 | 5,617,609 | 38.0 | 100.0 | 1.00 | 530,283 | 141 | 5,781 |
| SA6 | 4,060,963 | 44.0 | 100.0 | 0.06 | 1,053,606 | 56 | 4,053 |
| SB1 | 6,517,361 | 37.0 | 100.0 | 2.03 | 588,036 | 148 | 6,968 |
| SB8 | 5,712,906 | 38.0 | 99.67 | 2.15 | 750,902 | 124 | 5,710 |
| WA5 | 5,533,090 | 38.0 | 100.0 | 1.48 | 1,929,202 | 161 | 5,748 |
| WB3 | 3,864,777 | 41.0 | 100.0 | 0.23 | 147,213 | 136 | 3,988 |
| WB8 | 4,106,311 | 44.0 | 100.0 | 0.00 | 391,996 | 89 | 4,133 |

**Table S6.** Pairwise comparisons of sequenced genomes vs. type-strain genomes. Formula *d4* represents the sum of all identities found in HSPs divided by overall HSP length and is represented along with its corresponding confidence intervals (C.I. *d4*). Potential new species are specified by asterisks (*) in front of the isolates’ codes.

| **Isolate** | **Subject** | **d4**  **(%)** | **C.I. d4**  **(%)** | **G+C Difference (%)** |
| --- | --- | --- | --- | --- |
| SA1 | *Priestia megaterium* ATCC 14581 | 88.9 | [86.4 - 90.9] | 0.13 |
|  | WA5 | 73.2 | [70.2 - 76.1] | 0.09 |
| SA6 | *Bacillus subtilis* ATCC 6051 | 86.9 | [84.3 - 89.2] | 0.17 |
| SB1* | *Priestia aryabhattai* JCM 13839 | 68.6 | [65.6 - 71.4] | 0.96 |
|  | *Priestia megaterium* ATCC 14581 | 64 | [61.1 - 66.8] | 0.82 |
|  | *Rummeliibacillus pycnus* DSM 15030 | 25.9 | [23.6 - 28.4] | 2.36 |
| SB8 | *Niallia taxi* M5HDSG1-1T | 91.9 | [89.8 - 93.6] | 0.01 |
| WA5 | *Priestia megaterium* ATCC 14581 | 73.6 | [70.6 - 76.4] | 0.04 |
| WB3* | *Bacillus pumilus* ATCC 7061 | 63.4 | [60.5 - 66.2] | 0.84 |
|  | *Bacillus safensis* FO-36b | 41.9 | [39.4 - 44.5] | 0.78 |
|  | *Bacillus australimaris* NH7I_1 | 40.9 | [38.4 - 43.4] | 0.52 |
| WB8 | *Metabacillus indicus* LMG 22858 | 71.4 | [68.4 - 74.2] | 0.02 |

**Table S7.** Isolates genome availability on NCBI and their corresponding taxonomic identification according to TYGS analysis (phylogenetic placement and *d4* formula).

| **Sample** | **Isolation**  **Source** | **Taxonomy** | **BioProject** | **BioSample Accession** | **Genome Accession** |
| --- | --- | --- | --- | --- | --- |
| SB1 | Colhereiro sediment | *Priestia* sp. SB1 * | PRJNA1042624 | SAMN40379371 | JBBJSS000000000 |
| SB8 | Colhereiro sediment | *Niallia taxi* |  | SAMN40379372 | JBBJSR000000000 |
| SA1 | Araçá sediment | *Priestia megaterium* |  | SAMN40379373 | JBBJSQ000000000 |
| SA6 | Araçá sediment | *Bacillus subtilis* |  | SAMN40379374 | JBBJSP000000000 |
| WA5 | Araçá  water | *Priestia megaterium* |  | SAMN40379375 | JBBJSO000000000 |
| WB3 | Colhereiro water | *Bacillus* sp. WB3 * |  | SAMN40379376 | JBBJSN000000000 |
| WB8-II | Colhereiro water | *Metabacillus indicus* |  | SAMN40379377 | JBBJSM000000000 |

* Potential new species

**Table S8.** Antibiotic resistance genes (ARGs) identified in sequenced genomes using ABRICATE pipeline.

| **Isolate** | **ARG** | **Description** |
| --- | --- | --- |
| SA1 | *lsaB* | LsaB is an ABC-F subfamily protein expressed in *Staphylococcus sciuri*. It confers resistance to clindamycin. |
| SA6 | *rphB* | rphB is a rifampin phosphotransferase protein found in *Paenibacillus* sp. LC231 a strain of *Paenibacillus* isolated from Lechuguilla Cave NM USA. Confers resistance to rifamycin antibiotics specifically rifampin through rifampin inactivation. Described by Pawlowski et al. 2016. |
| SA6 | *bmr* | bmr is an MFS antibiotic efflux pump that confers resistance to multiple drugs including acridine dyes fluoroquinolone antibiotics chloramphenicol and puromycin |
| SA6 | *blt* | blt is an MFS efflux pump that confers resistance to multiple drugs such as rhodamine and acridine dyes and fluoroquinolone antibiotics |
| SA6 | *aadK* | aadK is a chromosomal-encoded aminoglycoside nucleotidyltransferase gene in B. subtilis and Bacillus spp. |
| SA6 | *ykkC* | ykkC is an SMR-type protein that is a subunit of the ykkCD efflux pump |
| SA6 | *ykkD* | ykkD is an SMR-type protein that is a subunit of the ykkCD efflux pump |
| SA6 | *mprF* | MprF is a integral membrane protein that modifies the negatively-charged phosphatidylglycerol on the membrane surface. This confers resistance to cationic peptides that disrupt the cell membrane including defensins. Additionally large-scale mutations causing loss of function of the gene result in increased susceptibility to daptomycin. |
| SA6 | *vmlR* | vmlR is an ABC-F ATPase ribosomal protection protein identified in Bacillus subtilus. Shown to confer resistance to lincomycin and streptogramin A virginiamycin. Described by Crowe-McAuliffe et al. 2018. |
| SA6 | *tmrB* | tmrB is an ATP-binding tunicamycin resistance protein found in Bacillus subtilis |
| SA6 | *lmrB* | lmrB is a chromosomally-encoded efflux pump that confers resistance to lincosamides in Bacillus subtilis |
| SA6 | *mphK* | A chromosomal macrolide phosphotransferase identified from Bacillus subtilis |
| SA6 | *rphC* | rifamycin-inactivating phosphotransferase RphC |
| WB3 | *BPU-1* | BPU-1 is a class D beta-lactamase found in Bacillus pumilus. |
| WB3 | *cat86* | cat86 is a chromosome-encoded variant of the cat gene found in Bacillus pumilus |
